# Stringing Bimetallic Metal–Organic Framework‐Derived Cobalt Phosphide Composite for High‐Efficiency Overall Water Splitting

**DOI:** 10.1002/advs.201903195

**Published:** 2020-01-23

**Authors:** Lulu Chai, Zhuoyi Hu, Xian Wang, Yuwei Xu, Linjie Zhang, Ting‐Ting Li, Yue Hu, Jinjie Qian, Shaoming Huang

**Affiliations:** ^1^ Key Laboratory of Carbon Materials of Zhejiang Province College of Chemistry and Materials Engineering Wenzhou University Wenzhou 325000 China; ^2^ State Key Laboratory of Structural Chemistry Fujian Institute of Research on the Structure of Matter Chinese Academy of Sciences Fuzhou 350002 China; ^3^ Chimie du solide et de l'énergie‐Collège de France 11 Place Marcelin Berthelot Paris 75005 France; ^4^ School of Materials Science and Chemical Engineering Ningbo University Ningbo 315211 China; ^5^ School of Materials and Energy Guangdong University of Technology Guangzhou 510006 China

**Keywords:** cobalt phosphide, metal–organic frameworks, nitrogen‐doped carbon nanotubes, overall water splitting

## Abstract

Water electrolysis is an emerging energy conversion technology, which is significant for efficient hydrogen (H_2_) production. Based on the high‐activity transition metal ions and metal alloys of ultrastable bifunctional catalyst, the hydrogen evolution reaction (HER) and oxygen evolution reaction (OER) are the key to achieving the energy conversion method by overall water splitting (OWS). This study reports that the Co‐based coordination polymer (ZIF‐67) anchoring on an indium–organic framework (InOF‐1) composite (InOF‐1@ZIF‐67) is treated followed by carbonization and phosphorization to successfully obtain CoP nanoparticles–embedded carbon nanotubes and nitrogen‐doped carbon materials (CoP‐InNC@CNT). As HER and OER electrocatalysts, it is demonstrated that CoP‐InNC@CNT simultaneously exhibit high HER performance (overpotential of 153 mV in 0.5 m H_2_SO_4_ and 159 mV in 1.0 m KOH) and OER performance (overpotential of 270 mV in 1.0 m KOH) activities to reach the current density of 10 mA cm^−2^. In addition, these CoP‐InNC@CNT rods, as a cathode and an anode, can display an excellent OWS performance with η_10_ = 1.58 V and better stability, which shows the satisfying electrocatalyst for the OWS compared to control materials. This method ensures the tight and uniform growth of the fast nucleating and stable materials on substrate and can be further applied for practical electrochemical reactions.

## Graphical abstract

A type of CoP embedded in carbon nanotubes and nitrogen‐doped carbon material calcined from a bimetallic metal–organic frameworks (MOF) precursor is designed and prepared by growing Co‐based MOFs on an indium–organic framework. The CoP incorporation can greatly promote the water splitting kinetics by the optimized catalyst of CoP‐InNC@CNT, thus the high electrocatalytic activity is achieved toward both the hydrogen evolution reaction and oxygen evolution reaction.

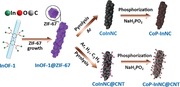

## 1 Introduction

Along with the population growth and the economic development, there is a growing concern about energy crises and climate change issues. In this context, the electrolysis of water is a promising and environmental friendly method to prepare clean, harmless, and sustainable H_2_ fuels on a large scale.^1^, ^2^, ^3^ Since the overall water splitting (OWS) process is different from the steam reforming of hydrocarbons, there is no emissions of any contaminants in the synthetic process.^4^ More importantly, the hydrogen is fabricated by the water electrolysis, which is of high purity and does not contain carbon monoxide, so it can be used directly to power the fuel cells without poisoning catalyst on the electrodes.^5^ In this regard, although it can be regarded as one powerful tool to simultaneously and efficiently generate hydrogen and oxygen,^6^, ^7^ the OWS process generally exhibits an energy‐consuming system with the high overpotential (η).^8^, ^9^ Currently, noble metal oxides about ruthenium or iridium (RuO_2_ and IrO_2_) for oxygen evolution reaction (OER) and metallic platinum (Pt) for hydrogen evolution reaction (HER), are still the standard catalysts for the practical OWS applications due to their high activity and stability, but it greatly limits their use in large‐scale applications owing to the high cost and scarce availability.^10^ Therefore, the electrocatalysts based on low‐cost, environmentally friendly, and earth‐rich transition metals must be rationally designed to greatly reduce the overpotential and efficiently improve the energy efficiency of the OWS system.^11^, ^12^, ^13^, ^14^

In recent years, transition metal phosphides (TMPs) are a critical class of compounds, which are mainly characterized by the low fabrication cost, high electrical conductivity, and electrochemical stability.^15^, ^16^, ^17^, ^18^, ^19^ Early studies have shown that cobalt phosphide exhibits an excellent hydrodesulfurization performance with a similar operating mechanism to HER, which has been recognized as an efficient catalysts for HER catalysts since then.^20^, ^21^, ^22^ Meanwhile, researches have also shown that TMPs can also catalyze in OER process.^23^ Among them, cobalt phosphide has received an extensive attention because these negatively charged phosphorous atoms can effectively capture protons and promote the release of H_2_. On the other hand, the positively charged cobalt cations may serve as hydroxyl receptors, while the negatively charged P centers facilitate the desorption of O_2_ molecules.^24^, ^25^, ^26^ Other works have revealed that the combination of CoP particles with the nanocarbon matrix, including carbon nanotubes (CNTs),^27^ graphene,^28^ and metal–organic frameworks (MOFs) derived porous carbon,^29^, ^30^ can further improve the electrochemical performance for the high specific surface area and electrical conductivity. In addition, Su and co‐workers reported two types of ternary hybrids (Co_2_P/Mo_2_C/Mo_3_Co_3_C@C and Ni/Ni_2_P/Mo_2_C@C) derived from two bimetallic MOFs of CoMo–MOF and NiMo–MOF, which render the outstanding bifunctional electrocatalytic properties in the processes of HER and OER.^31^ Luo and co‐workers publish a facile two‐step ZIF‐derived route to synthesize CoP nanoparticles (NPs) encapsulated in an ultrathin nitrogen‐doped porous carbon shell (CoP@NC), which can also serve as an outstanding electrocatalyst.^32^ Generally speaking, hierarchically porous MOF‐derived composites can effectively enhance the overall performance through synergistic configuration of each component.

**Scheme 1:**
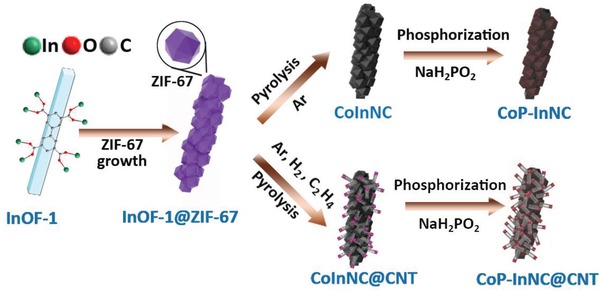
Schematic illustration of the synthetic process for sugar‐gourd‐like CoP‐InNC and CoP‐InNC@CNT composites.

Inspired by the above considerations, we have rationally designed the catalyst for the preparation of cobalt phosphide NPs embedded in carbon nanotubes and nitrogen‐doped carbon (CoP‐InNC@CNT) by growing Co‐based coordination polymer (ZIF‐67) on an indium–organic framework (InOF‐1) (**Scheme** **1**), which simultaneously demonstrates the high HER performance (overpotential of 153 mV in 0.5 m H_2_SO_4_ and 159 mV in 1.0 m KOH) and OER activity (overpotential of 270 mV in 1.0 m KOH) to reach the current density of 10 mA cm^−2^. In addition, by integrating its bifunctional catalytic performance, the CoP‐InNC@CNT/CC||CoP‐InNC@ CNT/CC device with only a small battery voltage of 1.58 V can be used to efficiently drive the OWS to a current density of 10 mA cm^−2^. Similarly, this CoP‐InNC@CNT‐based device exhibits an excellent stability for OWS. In this case, the current findings indicate that the rational design of hierarchically bimetallic MOF‐derived Co‐based phosphide composite can be potentially applied in the practical OWS performance.

## 2 Results and Discussion

### 2.1 Material Characterization of the CoP‐InNC@CNT Microrods

**Figure 1:**
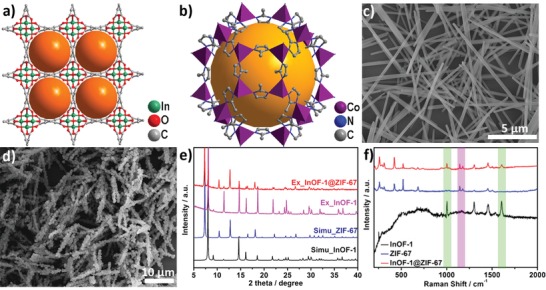
Crystal structures of a) InOF‐1 and b) ZIF‐67; SEM images of c) InOF‐1 and d) InOF‐1@ZIF‐67 rods; PXRD patterns e) and Raman spectra f) of InOF‐1@ZIF‐67 microrods.

The detailed synthetic method of the CoP‐InNC@CNT catalysts is well illustrated in Scheme 1. First, we obtain an indium–organic skeleton of [In_2_(OH)_2_(BPTC)]⋅6H_2_O (InOF‐1, H_4_BPTC = biphenyl‐3,3ʹ,5,5ʹ‐tetracarboxylic acid) as a template by a typical solvothermal reaction. In the InOF‐1 crystal structure (**Figure** **1**a), each BPTC^4−^ linker is connected to eight independent In(III) centers by its four carboxylate groups, which are in 6‐coordinated octahedron geometry composed of four carboxylate oxygen atoms from four deprotonated BPTC^4−^ ligands together with two oxygen atoms from two hydroxyl groups (–OH).^33^ The BPTC^4−^ ligands cooperate with the In(III) centers to generate the tetragonal cylindrical channels in the microporous InOF‐1, which exhibits a microrod morphology enabling it to be used as an excellent nanosubstrate. After that, ZIF‐67 polyhedra anchored on the InOF‐1 rods can be rapidly formed by the deprotonation coordination reaction of Hmim with Co^2+^ ions.^34^ In the subsequent growth process, they will gradually and successfully form the precursor of InOF‐1@ZIF‐67 (Figure 1b). The scanning electron microscope (SEM) shows that the InOF‐1 rods have a diameter of 200 nm with a smooth surface (Figure 1c), while the SEM image of the InOF‐1@ZIF‐67 composite reveals a typical core–shell morphology, where ZIF‐67 is uniformly coated on InOF‐1 rods to form a 3D superstructure (Figure 1d; Figures S1 and S2, Supporting Information). The powder X‐ray diffraction (PXRD) patterns of pure InOF‐1 and InOF‐1@ZIF‐67 composites are completely consistent with the simulated MOFs. In the range of 5°–15° in PXRD, the peaks of 2θ = 8.1°, 13.3°, 14.6° and 7.4°, 10.4°, 12.7° are attributed to the peaks of InOF‐1 and ZIF‐67, respectively, which proves two crystal structures coexisting in InOF‐1@ZIF‐67 composites (Figure 1e). To further verify the successful loading of ZIF‐67 particles on InOF‐1 rods, we have also performed Raman analysis at the 200–2000 cm^−1^ range (Figure 1f). It is obvious that both the characteristic peaks of ZIF‐67 (1145, 1177 cm^−1^) and InOF‐1 (1004, 1605 cm^−1^) are observed in InOF‐1@ZIF‐67. Fourier transform infrared (FT‐IR) spectroscopy also characterizes the same results (Figure S3, Supporting Information).

**Figure 2:**
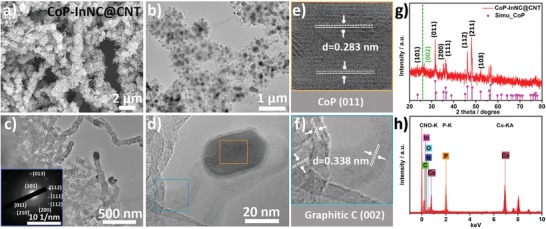
a) SEM; b,c) TEM images at different magnification of CoP‐InNC@CNT rods, inset in (c): SAED image; e,f) HR‐TEM images of the selected area in (d), respectively; g) PXRD pattern and h) EDS spectrum of CoP‐InNC@CNT rods.

Then, the thermogravimetric analyse (TGA) demonstrates that InOF‐1, ZIF‐67, and InOF‐1@ZIF‐67 have good thermal stability in Figure S4 in the Supporting Information. After in situ pyrolysis treatment of InOF‐1@ZIF‐67 at 800 °C in different flowing gases (Ar, Ar/H_2_/C_2_H_4_), CoInNC and CoInNC@CNT samples are conveniently obtained, which are also fully interpreted in Figures S5 and S6 in the Supporting Information. By comparing CoInNC and CoInNC@CNT catalysts, the key to the formation of carbon nanotubes is the effective catalytic effect of Co nanoparticles.^35^ It is clearly shown that the rough surface of CoInNC@CNT is inherently anchored with numerous CNTs. After that, the NaH_2_PO_2_ powders and CoInNC or CoInNC@CNT materials are simultaneously placed in two separate quartz boats, and then heated at different temperatures under the protection of Ar atmosphere to successfully convert the Co NPs embedded in the porous carbon layer into CoP NPs (**Figure** **2**; Figure S7, Supporting Information).

**Figure 3:**
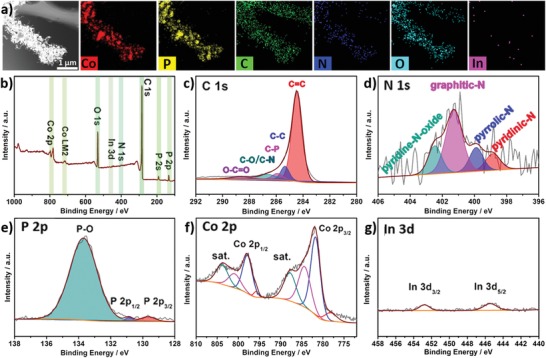
a) The mapping element images, b) full XPS survey, c) C 1s, d) N 1s, e) P 2p, f) Co 2p, and g) In 3d deconvoluted XPS spectra for CoP‐InNC@CNT rods.

As clearly depicted in the SEM and TEM images of CoP‐InNC@CNT (Figure 2a,b), it is shown that the prepared CoP‐InNC@CNT composite features a rod‐like structure in which CNTs are rooted at the surface and CoP NPs are encapsulated by graphitic carbons on the tips of CNTs. Figure 2c further shows that the single CNT with a diameter of ≈50 nm is multiwalled. Meanwhile, as shown the selected area electron diffraction (SAED) image, it reveals that the main diffraction rings match with the CoP‐InNC@CNT (inset in Figure 2c). The high‐resolution TEM (HR‐TEM) images of the CoP‐InNC@CNT exhibit the distinct fringe spacing of 0.283 nm corresponding to the (011) lattice plane of CoP, and a *d*‐spacing of ≈0.338 nm of unique lattice fringe (Figure 2d–f), which is consistent with the (002) diffraction plane of graphitic carbon,^36^ respectively. In the PXRD pattern of CoP‐InNC@CNT (Figure 2g), the diffraction peaks located at 23.6°, 31.6°, 35.3°, 36.3°, 46.2°, 48.1°, and 52.2° can be perceived, corresponding to the (101), (011), (200), (111), (112), (211), and (103) facets of CoP (PDF#65‐2593). Meanwhile, a broad peak appeared at 25.7° can be attributable to the (002) lattice plane of graphitic carbon. Furthermore, energy dispersive X‐ray spectroscopy (EDS) spectrum displays the presence of C, N, O, In, Co, and P elements (Figure 2h). Furthermore, high‐angle annular dark field scanning TEM (HAADF‐STEM) and the related element mapping analysis exhibit that the elements of Co, P, C, N, and O are uniformly distributed in the entire architecture, while the In element is less present throughout the region due to the evaporation at high temperature (**Figure** **3**a), which is in good accordance with XPS analysis below.^37^ In this context, the corresponding C, N, P, O, Co, and In contents are obtained by the EDX (Table S1, Supporting Information), which exhibit the presence of well distributed C, N, P, O, Co, and In in the CoP‐InNC@CNT. Among that the Co and In contents can be confirmed by elemental analysis. The metal Co and In contents are calculated to be 1.87% and 0.01% in the as‐pyrolyzed CoP‐InNC@CNT catalyst, respectively, which is in agreement with HAADF‐STEM image.

To further investigate the types and chemical states of the surface element, X‐ray photoelectron spectroscopy (XPS) is utilized to characterize the elements of C, N, P, O, Co, and In for CoInNC, CoInNC@CNT, CoP‐InNC, and CoP‐InNC@CNT (Figure 3; Figures S10–S13, Supporting Information). The XPS survey spectrum (Figure 3b) displays the C, N, In, Co, and P elements together with the absorbed O_2_/CO_2_ from the air or a small amount of O element that may be generated by the remaining oxygen‐containing groups in the precursor.^38^ For the C 1s spectrum, the band is deconvoluted into five peaks (Figure 3c), in which the peaks of 284.5 and 285.3 eV are indexed to the sp^2^ C=C bond and the sp^3^ C—C bond in the C 1s spectrum, while the binding energy peaks at 286.0 eV correspond to the C—P matrix. In addition, the peaks at 286.6 and 288.7 eV suggest the formation of C—O/C—N groups and the —COO bonds, respectively.^24^ Figure 3d corroborates that the four major peaks with the binding energy are 398.8, 399.9, 401.2, and 402.5 eV, which are assignable to pyridinic nitrogen, pyrrolic nitrogen, graphitic nitrogen, and pyridine‐N‐oxide in the high‐resolution N 1s XPS, respectively.^39^ In addition, the P 2p spectrum is deconvoluted into three accurately separated peaks (Figure 3e), where the doublet binding energy peaks appeared at 129.6 and 130.8 eV can be associated with P 2p_3/2_ and P 2p_1/2_ of the generation of Co—P species, respectively, and the binding energy of 133.7 eV is ascribed to the P—O groups, which are possibly derived from cobalt phosphates by superficial oxidation.^32^ There are two distinct areas in the Co 2p spectrum: the Co 2p_3/2_ area of the low binding energy region and the Co 2p_1/2_ area of the high binding energy region (Figure 3f). For CoP‐InNC@CNT rods, the prominent peaks that located in Co 2p_3/2_ and Co 2p_1/2_ region at 777.8/795.3, 781.9/797.6, 785.3/800.7, and 787.9/803.7 eV can be reasonably attributed to residual metallic Co, the oxidized Co^2+^/Co^3+^, the Co—N bond, and Co satellite peaks (sat.), respectively.^40^, ^41^ Then, the deconvoluted high‐resolution O 1s XPS spectrum of CoP‐InNC@CNT can be compared with CoInNC, CoInNC@CNT, and CoP‐InNC as control samples by fitting multiple peaks. As shown in Figure S13 in the Supporting Information, the lower energy peaks of 530.3 ± 0.2 eV can be attributed to the Metal–O group (Co–O and In–O). The peaks at 531.3 ± 0.3 and 533.4 ± 0.3 eV are usually related to the O species in the C—O bonds and the specific chemisorbed oxygen and/or adsorbed H_2_O on the surface of the materials. The difference is that the peak at the binding energy of 532.2 ± 0.1 eV is assigned to the P—O bond in CoP‐InNC@CNT and CoP‐InNC. Finally, the high‐resolution In 3d XPS spectrum corroborates two small peaks at 445.4 eV (In 3d_5/2_) and 452.8 eV (In 3d_3/2_) (Figure 3g), which are indexed to the In cation with a valence of +3 from the residual indium oxide.^42^, ^43^

**Figure 4:**
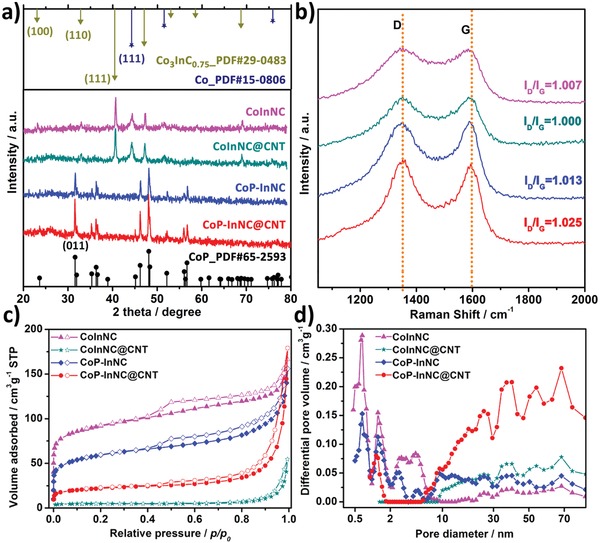
a) PXRD patterns; b) Raman spectra; c) Nitrogen sorption isotherms at 77 K (closed, adsorption; open, desorption); and d) the corresponding pore size distribution curves of CoInNC, CoInNC@CNT, CoP‐InNC, and CoP‐InNC@CNT rods.

The detailed structural characterization of CoInNC, CoInNC@CNT, CoP‐InNC, and CoP‐InNC@CNT rods is identified by PXRD pattern (**Figure** **4**a). Both CoInNC and CoInNC@CNT rods display four diffraction peaks of 23.0°, 32.8°, 40.4°, and 44.2°, exactly corresponding to (100), (110), (111) planes of Co_3_InC_0.75_ (PDF#29‐0483) and (111) plane of Co (PDF#15‐0806), respectively. Meanwhile, the diffraction peaks of the InOF‐1 and ZIF‐67 precursors are not detected, which clearly indicate that the structures of MOFs have been completely and thermally decomposed after the carbonization treatment, revealing that the indium species can facilitate the formation of heterometallic carbides. On the other hand, the PXRD patterns of CoP‐InNC and CoP‐InNC@CNT rods are matched well with the lattice plane of CoP (PDF#65‐2593). In contrast, the clear Co diffraction peak at 44.2° is not observed in Figure 4a, which demonstrates that most of all metallic Co NPs at the high‐temperature phosphatization have been converted into the corresponding CoP NPs. Then, the structure of carbon in the composite is investigated by using the Raman spectrum in Figure 4b, in which the two peak values at 1358 and 1590 cm^−1^ are assignable to the two carbon bands of the D and G bands, which correspond to the defect density and the graphitization, respectively. The intensity ratios (*I*_D_/*I*_G_) of the Raman‐active D band and the G band of CoInNC, CoInNC@CNT, CoP‐InNC, and CoP‐InNC@CNT are 1.007, 1.000, 1.013, and 1.025, respectively, indicating a decreased degree of graphitization with the prolongated preparation time. The surface area and pore structure information on the CoInNC, CoInNC@CNT, CoP‐InNC, and CoP‐InNC@CNT catalysts are investigated by N_2_ sorption at 77 K, and the structural properties of all materials are comprehensively summarized in Table S2 in the Supporting Information. In Figure 4c,d, as compared to CoP‐InNC (211 m^2^ g^−1^) and CoInNC (335 m^2^ g^−1^), CoP‐InNC@CNT (79 m^2^ g^−1^), and CoInNC@CNT (19 m^2^ g^−1^) electrocatalysts have a smaller Brunauer–Emmett–Teller (BET) specific surface area mainly owing to the growth of CNTs.^44^ The CoInNC and CoPInNC catalysts exhibit type I isotherms and maintain the microporous structure of InOF‐1@ZIF‐67 (Figure S9, Supporting Information), indicating the existence of micropores, mesopores and macropores of these MOF‐derived materials. However, the type IV isotherms are observed with distinct hysteresis loop for the CoInNC@CNT and CoP‐InNC@CNT catalysts, which corroborates that mesoporous structure is abundant. In this context, the hierarchical porosity of the CoP‐InNC@CNT catalyst will facilitate the electrocatalytic charge and mass transport in the following test.

### 2.2 Hydrogen Evolution Reaction of the CoP‐InNC@CNT Microrods

**Figure 5:**
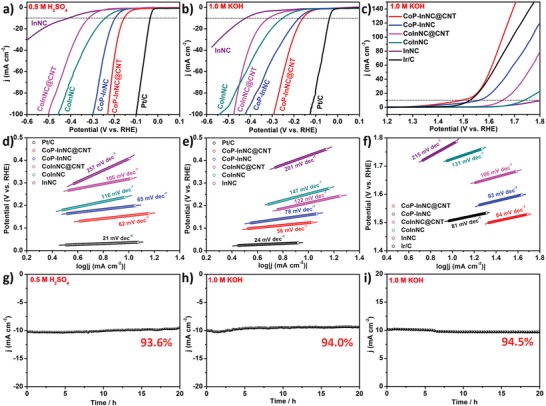
a–c) LSV curves and d–f) their corresponding Tafel slopes of CoP‐InNC@CNT and control samples in 0.5 m H_2_SO_4_, 1.0 m KOH for HER, and 1.0 m KOH for OER, respectively. g–i) The time‐dependent (*i–t*) of current density curves of CoP‐InNC@CNT in 0.5 m H_2_SO_4_, 1.0 m KOH for HER, and 1.0 m KOH for OER, respectively.

The catalytic HER activity of CoP‐InNC@CNT is investigated using a standard 3‐electrode system in 0.5 m H_2_SO_4_ and 1.0 m KOH. To avoid platinum contamination, a graphite rod as the counter electrode is used in our tests.^45^ For comparison, a series of comparative catalysts are also tested, including CoInNC, CoInNC@CNT, CoP‐InNC, InNC catalysts (Figure S8, Supporting Information) and commercial 20% Pt/C. Linear sweep voltammetry (LSV) curves of all samples are detected at ambient temperature by using a 5 mV s^−1^ scan rate (**Figure** **5**a,b). Sure enough, the 20% Pt/C catalyst possesses the highest activity as a control in the HER process and the overpotentials at the current density of 10 mA cm^−2^ (η_10_) are small of 35 mV in 0.5 m H_2_SO_4_ and 37 mV 1.0 m KOH. Compared with CoP‐InNC (195 and 177 mV), CoInNC (241 and 252 mV), CoInNC@CNT (318 and 316 mV), and InNC (409 and 421 mV), the HER catalytic activity for CoP‐InNC@CNT catalyst corroborates a higher HER activity with η_10_ = 153 and 159 mV in 0.5 m H_2_SO_4_ and 1.0 m KOH solution. Meanwhile, the corresponding Tafel slopes of the CoP‐InNC@CNT catalyst (62 and 56 mV dec^−1^) are significantly lower than those of CoP‐InNC (65 and 78 mV dec^−1^), CoInNC (116 and 147 mV dec^−1^), CoInNC@CNT (106 and 122 mV dec^−1^), and InNC (257 and 201 mV dec^−1^) in the electrolyte solution of 0.5 m H_2_SO_4_ and 1 m KOH, respectively (Figure 5b,e). These results indicate that electrochemical hydrogen desorption on the CoP‐InNC@CNT catalyst is a rate‐determining step following the Volmer–Heyrovský pathway and is conducive to the HER activity.^46^ Although the catalytic activity of Pt/C is significantly higher than that of the CoP‐InNC@CNT rods, it is competitive compared with the nonprecious metal‐based catalyst in the acidic and alkaline electrolytes (Table S3, Supporting Information). In addition, the large electrochemically active surface area (ECSA) of the CoP‐InNC@CNT rods is revealed by CV‐based electrochemical double layer capacitance measurements (*C*_dl_) at the range of 20–200 mV s^−1^ in both 0.5 m H_2_SO_4_ solution and 1 m KOH solution (Figures S14, S15, and S17a,b, Supporting Information), suggesting the more active sites in CoP‐InNC@CNT rods for the HER performance. In Figures S18 and S19 in the Supporting Information, the charge transfer resistances (*R*_ct_) of the CoP‐InNC@CNT catalysts are 3.06 Ω in the electrolyte of 0.5 m H_2_SO_4_ and 2.73 Ω in the electrolyte of 1.0 m KOH, respectively, which are obviously smaller than that of CoP‐InNC (3.47 and 4.01 Ω), CoInNC (7.06 and 4.56 Ω), CoInNC@CNT (5.47 and 4.07 Ω), and InNC (4.21 and 4.30 Ω). Generally, a smaller *R*_ct_ value indicates the existence of a faster charge transfer rate during the reaction, which subsequently implies that the CoP‐InNC@CNT catalyst owns the highest electrocatalytic reaction efficiency among them. The above results prove that the positive effect of phosphorization is important to HER electrocatalytic activity. In addition to the study of activity and mechanism, the durability experiment of the CoP‐InNC@CNT catalyst at the electrolytes of 0.5 m H_2_SO_4_ and 1.0 m KOH for HER is also tested to confirm the feasibility in practical applications (Figure 5g,h). The *i–t* curves clearly show that the CoP‐InNC@CNT catalyst can continuously drive a current density around 10 mA cm^−2^ and decay slowly within 20 h, showing good stability against HER.

### 2.3 Oxygen Evolution Reaction of the CoP‐InNC@CNT Microrods

To implement the OER electrocatalytic activity, CoP‐InNC@CNT samples coated on the GCE electrode then are assessed in 1.0 m KOH electrolyte for an OER electrocatalytic activity. The CoInNC, CoInNC@CNT, CoP‐InNC, InNC catalysts, and commercial Ir/C are also investigated under the same conditions for comparison. LSV curves of all catalysts are tested by using a 5 mV s^−1^ scan rate (Figure 5c). Compared with CoP‐InNC (η_10_ = 330 mV), CoInNC (η_10_ = 507 mV), CoInNC@CNT (η_10_ = 423 mV), InNC (η_10_ = 582 mV), and commercial Ir/C (η_10_ = 295 mV), the as‐obtained CoP‐InNC@CNT catalyst possesses the highest electrocatalytic OER performance with a lower η_10_ of 270 mV comparable to these reported materials in the literature (Table S3, Supporting Information). The overpotentials of CoP‐InNC@CNT and CoInNC@CNT are lower than those of CoP‐InNC and CoInNC, respectively, demonstrating that both CoP and CNT can optimize the catalytic performance efficiently. In Figure 5f, the corresponding Tafel slope of the CoP‐InNC@CNT catalyst (84 mV dec^−1^) is also smaller than those of CoP‐InNC (93 mV dec^−1^), CoInNC (131 mV dec^−1^), CoInNC@CNT (106 mV dec^−1^), and InNC (215 mV dec^−1^) in 1.0 m KOH, which suggests the fastest ion diffusion path can accelerate the OER rate of the CoP‐InNC@CNT catalyst. Meanwhile, the *C*_dl_ values of CoP‐InNC@CNT (20.66 mF cm^−2^), CoP‐InNC (20.22 mF cm^−2^), CoInNC (28.16 mF cm^−2^), CoInNC@CNT (17.68 mF cm^−2^), InNC (2.72 mF cm^−2^), and Ir/C (21.56 mF cm^−2^) are shown in Figures S16 and S17c in the Supporting Information. The CoP‐InNC@CNT catalyst displays shows a good *C*_dl_ value, demonstrating that it owns more catalytic active sites. In addition, the excellent catalytic properties of CoP‐InNC@CNT catalyst can be attributed to its abundant reaction sites, such as Co–N, Co–P, CNT conductivity, and N‐doped carbon, and the porous nanostructure with high specific surface area. Finally, the long‐term stability measurement for CoP‐InNC@CNT is also obtained, whose current density loss percentage in the *i–t* curve of CoP‐InNC@CNT is 94.5% after 20 h, indicating an excellent stability in 1.0 m KOH solution (Figure 5i).

### 2.4 Overall Water Splitting of the CoP‐InNC@CNT Microrods

**Figure 6:**
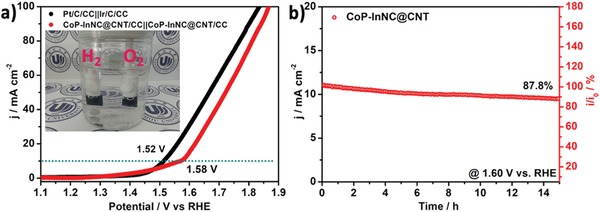
a) LSV curves of an overall water splitting in a two‐electrode configuration. The inset is the digital photograph of H_2_ and O_2_ bubbles. b) Time‐dependent current density curves for CoP‐InNC@CNT/CC||CoP‐InNC@CNT/CC at a fixed potential for 15 h.

Compared with other control samples, the above experiment confirms that CoP‐InNC@CNT catalysts have higher catalytic activity for both HER in 0.5 m H_2_SO_4_ and 1.0 m KOH solution, and OER in 1.0 m KOH electrolyte. In view of HER and OER electrocatalytic properties, an electrolyzer is fabricated for the OWS test by using CoP‐InNC@CNT as the anode as well as the cathode on carbon cloth in the 1.0 m KOH electrolyte. Compared to the Pt/C/CC||Ir/C/CC appliance (1.52 V), our hand‐made CoP‐InNC@CNT/CC||CoP‐InNC@CNT/CC device only requires a small battery voltage of 1.58 V to drive the OWS to fully achieve a current density of 10 mA cm^−2^ (**Figure** **6**a). During electrolysis, both the cathode (H_2_) and the anode (O_2_) generate a large number of bubbles (Movie S1, Supporting Information), whose performance of the CoP‐InNC@CNT catalyst is superior to the most reported non‐noble metal electrocatalysts (Table S4, Supporting Information). Additionally, the device of CoP‐InNC@CNT exhibits the high durability for the OWS when the cell voltage is set to be 1.60 V, in which CoP‐InNC@CNT gradually decays within a period of 15 h and the retention rate is as high as 87.8%, indicating that CoP‐InNC@CNT has a satisfactory stability. In addition, SEM and TEM images indicate that the original morphology of the CoP‐InNC@CNT catalyst exhibits almost no change after a long‐term OWS electrolysis for 15 h (Figure S20a–c, Supporting Information). In the HR‐TEM image (Figure S20d, Supporting Information), the distinct fringe spacing of 0.286 nm is still consistent with the (011) diffraction plane of CoP (PDF#65‐2593). Meanwhile, the analysis of PXRD pattern (Figure S20e, Supporting Information) also demonstrates that the characteristic peaks of CoP after electrochemical OWS testing for 15 h are well preserved, indicating the good stability of the CoP‐InNC@CNT catalyst, which could be due to the CNT protection to avoid the CoP oxidation and/or decomposition. Therefore, the porous structure of CoP‐InNC@CNT rods can enhance charge transfer and improve electrocatalytic activity for the OWS. Thanks to the hierarchical MOF system, the special porous structure, high conductivity, fast electron transfer rate, and abundant active sites corresponding to the efficient synergetic interactions, the CoP‐InNC@CNT microrod benefits the excellent electrocatalytic performance and long‐term stability.

## 3 Conclusion

In summary, we have designed and prepared a type of the catalyst of CoP embedded in carbon nanotubes and nitrogen‐doped carbon material calcined from a bimetallic MOF precursor by growing Co‐based MOFs on an indium–organic framework. The CoP incorporation can greatly promote the water splitting kinetics by the optimized catalyst of CoP‐InNC@CNT, thus the high electrocatalytic activity is achieved towards both the HER in 0.5 m H_2_SO_4_ and 1.0 m KOH and OER in 1.0 m KOH. Specifically, the CoP‐InNC@CNT catalyst only requires the η_10_ as low as 153, 159, and 270 mV to drive HER and OER, respectively. In addition, the CoP‐InNC@CNT/CC||CoP‐InNC@CNT/CC appliance only requires a η_10_ of 1.58 V to drive the OWS with good stability. In our work, the bimetallic MOF‐derived CoP‐InNC@CNT material can not only act as a high‐performance OWS catalyst, but also initiate a strategy to synthesize CoP‐loaded nitrogen‐doped carbon composite with fine structure. Furthermore, the present work can be readily extended to offer the cost‐effective, energy‐efficient, and bifunctional electrocatalysts for performance enhancements of metal phosphide electrodes.

## 4 Experimental Section

##### Preparation of InOF‐1 ([In_2_(OH)_2_(BPTC)]·6H_2_O)

A mixture of In(NO_3_)_3_·xH_2_O (0.10 mmol, 30 mg), H_4_BPTC (0.05 mmol, 15 mg), and CTAB (0.01 mmol, 5 mg) in *N*,*N*‐dimethylformamide (3 mL) and H_2_O (3 mL) with the additional 0.1 mL HNO_3_ and 0.1 mL TEA was sonicated and placed in a 35 mL pressure tube, which was held at 140 °C for 30 min, and then cooled down to ambient temperature. By thorough washing, the InOF‐1 microrods were successfully collected from the solvents of DMF and EtOH several times at a centrifugal speed of 10 000 rpm, and then dried under vacuum at 85 °C. After drying, InOF‐1 powder (≈20 mg) was obtained with a yield of ≈57% based on initial organic ligands.

##### Preparation of Sugar‐Gourd‐Like InOF‐1@ZIF‐67 Rods

Typically, 10 mg of InOF‐1 microrods and 35 mg of PVP were well dispersed in 5 mL of MeOH, then Co(NO_3_)_2_∙O(_2_O (0.34 mmol, 99.6 mg) was dissolved in the above solution and sufficiently stirred for 1 h to form the A solution, while the B solution is consisted of Hmim (1.86 mmol, 153 mg) dissolved in 5 mL of MeOH. Then the mixture with A and B solution was stirred for 20 min after cooling at 70 °C and the precipitate was collected by centrifugation (9500 rpm, 3 min), then rinsed with EtOH solvent three times, and dried under vacuum overnight at 85 °C to obtain sugar‐gourd‐like InOF‐1@ZIF‐67 rods.

##### Preparation of CoIn‐NC and CoIn‐NC@CNT

CoIn‐NC and CoIn‐NC@CNT products were synthesized by the direct pyrolysis of InOF‐1@ZIF‐67 rods. The as‐prepared microrods in a quartz boat were transferred into a temperature‐programmed tube furnace, then pyrolyzed at a high temperature of 800 °C for 2 h under Ar (100 sccm) atmosphere to obtain CoIn‐NC, and under Ar (100 sccm), H_2_ (100 sccm), C_2_H_4_ (5.5 sccm) atmosphere to prepare CoIn‐NC@CNT, respectively. For the InNC tubes, the precursor with initial InOF‐1 rods was only replaced, which was then treated at the same carbonization environment as mentioned above.

##### Preparation of CoP‐InNC and CoP‐InNC@CNT

CoIn‐NC (≈80 mg) was transferred to the above temperature‐programmed tube furnace and also calcinated at a high temperature of 900 °C (3 h) in an Ar atmosphere (50 sccm). Meanwhile, NaH_2_PO_2_ samples (≈800 mg) were calcined at a low temperature of 300 °C for 3 h in the other upstream of the quartz tube by another isolated furnace (Scheme S1, Supporting Information). The obtained black sample was termed as CoP‐InNC. For the CoP‐InNC@CNT, the precursor of CoIn‐NC@CNT was also annealed by the same phosphatization reaction as CoP‐InNC treatment above.

##### Electrochemical Measurement

A standard 3‐electrode system (3 cm of glassy carbon electrode (GCE), Ag/AgCl, and graphite rod as the working, reference, and counter electrode, respectively) was used in this experiment. HER and OER tests for all samples were acquired at the Autolab workstation (Metrohm, Swiss) and/or CHI760E electrochemical workstation (CH Instruments, Shanghai). The detailed preparation of working electrode was narrated as below: 5 mg of catalyst was dissolved in 500 µL of mixture solvent (*V*_EtOH_:*V*_water_ = 9:1) and 20 µL of Nafion (5%), which was then ultrasonicated for 3 h. Thereafter, 10 µL of catalyst droplet was applied to the surface of the GCE with a micropipette and allowed to dry naturally (the loading amount = 0.35 mg cm^−2^). The LSV curves were investigated and recorded under the condition of a scan rate of 5 mV s^−1^, while all electrochemical impedance spectroscopy (EIS) measurement wasrecorded at the open‐circuit potential in the frequency range of 10^−2^–10^6^ Hz with an amplitude of 5 mV. In this work, all Ag/AgCl potentials were uniformly corrected to the standard reversible hydrogen electrode (RHE) without iR correction by pH calibration by the following equation: *E*_(RHE)_ = *E*_(Ag/AgCl)_ + 0.197 + 0.059 × pH. The overall water splitting tests were further detected in 1 m KOH electrolyte by using a two‐electrode system in which two modified catalysts were employed as both the cathode and the anode at the same time.

## Conflict of Interest

The authors declare no conflict of interest.

## Supporting information

Supporting Information

Supplemental Video 1
