# Supplementary material for: Stringing Bimetallic Metal–Organic Framework‐Derived Cobalt Phosphide Composite for High‐Efficiency Overall Water Splitting
Source: Adv Sci (Weinh). 2020 Jan 23;7(5):1903195. doi: 10.1002/advs.201903195 (PMC7055562; doi:10.1002/advs.201903195)
Supplement: Supplementary file 1 — Supporting Information [file ADVS-7-1903195-s001.pdf]

Copyright WILEY-VCH Verlag GmbH & Co. KGaA, 69469 Weinheim, Germany, 2020.

Supporting Information

for *Adv. Sci.,* DOI: 10.1002/advs.201903195

Stringing Bimetallic Metal–Organic Framework-Derived Cobalt Phosphide Composite for High-Efficiency Overall Water Splitting

Lulu Chai, Zhuoyi Hu, Xian Wang, Yuwei Xu, Linjie Zhang, Ting-Ting Li, Yue Hu, Jinjie Qian, and Shaoming Huang**

Copyright WILEY-VCH Verlag GmbH & Co. KGaA, 69469 Weinheim, Germany, 2019.

Supporting Information

Stringing Bimetallic MOF-derived Cobalt Phosphide Composite for High-Efficiency Overall Water Splitting

Lulu Chai, Zhuoyi Hu, Xian Wang, Yuwei Xu, Linjie Zhang, Ting-Ting Li, Yue Hu, Jinjie Qian, and Shaoming Huang**

Chemicals and materials.

All chemicals are reagent grade and used without processing unless otherwise described. Indium nitrate hydrate ($In(NO_3)_3{\cdot}xH_2O$, 99.9%, Aladdin), cobalt nitrate hexahydrate ($Co(NO_3)_2{\cdot}6H_2O$, 99%, Aladdin), biphenyl-3,3',5,5'-tetracarboxylic acid (H_4BPTC, 98.0%, Jinan Henghua Technology Company), 2-Methylimidazole (Hmim, 98%, Aladdin), hexadecyl trimethyl ammonium bromide (CTAB, 99%, Aladdin), polyvinylpyrrolidone (PVP, ~58000, Aladdin), platinum on carbon (Pt/C, 20%, Aladdin), Iridium on carbon (Ir/C, 10%, Aladdin), N,N-Dimethylformamide (DMF, 99.5%, Aladdin), de-ionized water (18 MΩ), methanol (MeOH, 99.5%, Aladdin), ethanol (EtOH, 95%, Aladdin), and triethylamine (TEA, 99%, Aladdin).

Physical measurement.

The SEM image is acquired by using a model JSM-6700F of a field emission scanning electron microscope operated at 10 kV. The related data of transmission electron microscope (TEM) and energy dispersive X-ray spectroscopy (EDS) are analyzed under the JEOL JEM-2100F microscope (200 kV). The powder X-ray diffraction (PXRD) patterns are collected on a Bruker D8 Advance at 40 kV and 40 mA with Cu Kα radiation (λ=0.154 nm). Thermogravimetric analysis (TGA) is implemented under a flowing N_2 atmosphere by using a NETZSCH STA 449C unit. Raman spectrometer is investigated on LabRAM HR Evolution from the 532 nm line of an Ar-ion laser. X-ray photoelectron spectroscopy (XPS) is recorded on a Thermo Scientific ESCALAB 250. Fourier transform infrared spectroscopy (FT-IR) spectra are carried on in the model of PerkinElmer Frontier MIR. N_2 sorption/desorption isotherms are analyzed at 77 K with Micromeritics ASAP 2020 surface-area analyzer.

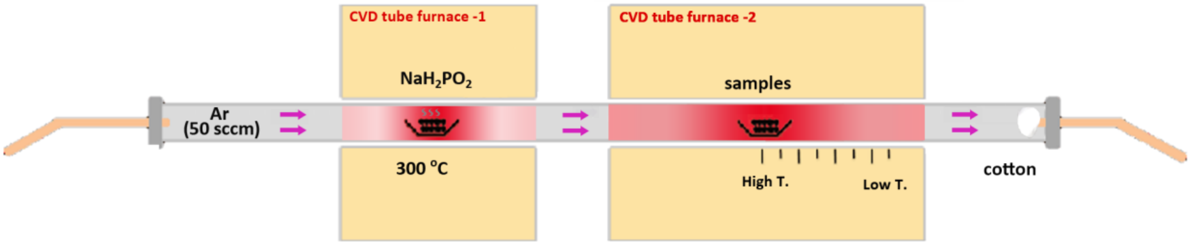

Scheme S1. Schematic diagram of the phosphating process with two seperate tube furnaces.

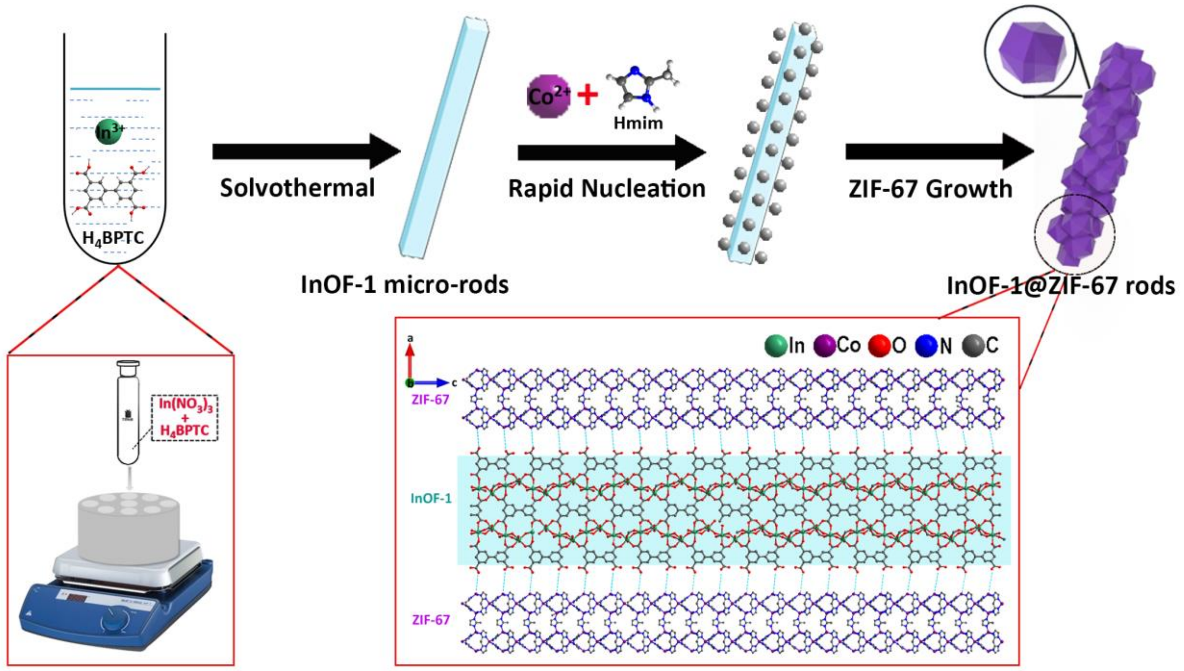

Figure S1. Schematic diagram of InOF-1@ZIF-67 mic-rods.

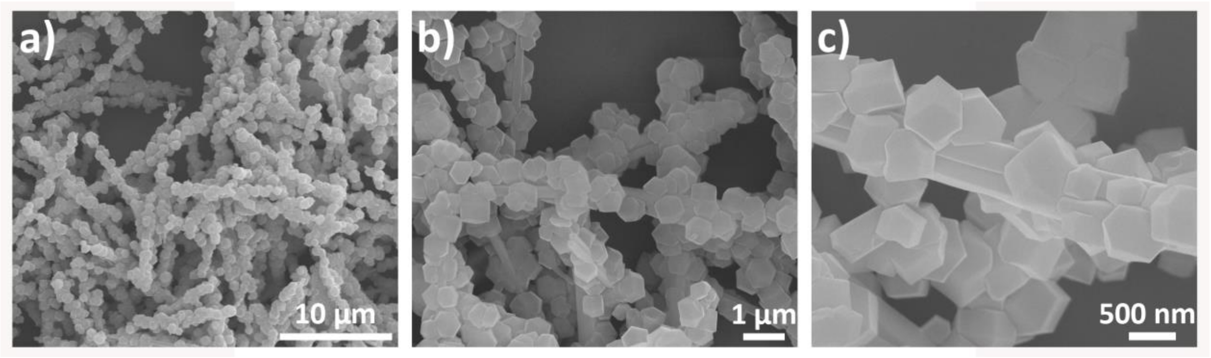

Figure S2. High-magnification SEM images (a, b, and c) of InOF-1@ZIF-67 mic-rods.

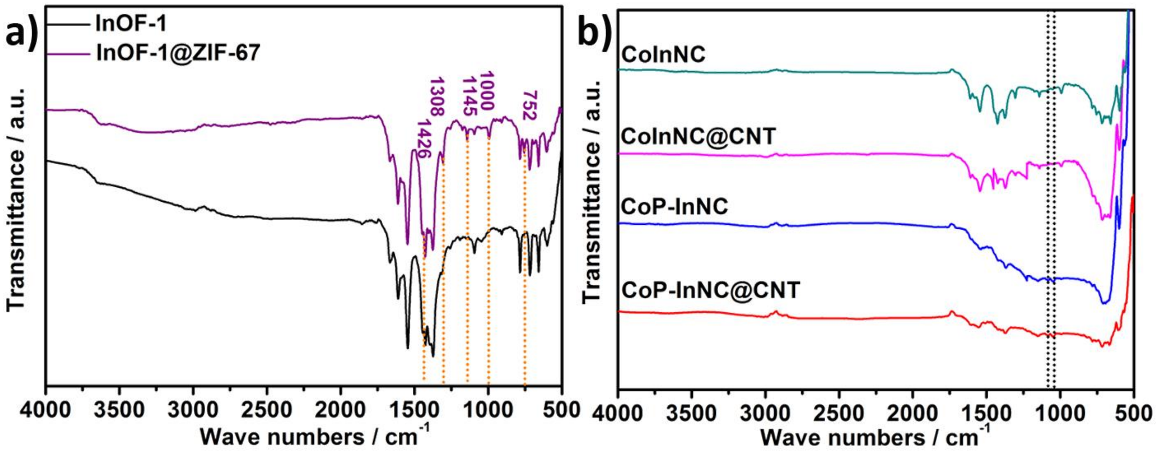

Figure S3. (a-b) FT-IR spectroscopy of InOF-1, InOF-1@ZIF-67, CoInNC, CoInNC@CNT, CoP-InNC, and CoP-InNC@CNT samples.

To further clarify the conversion between chemical components, the chemical bonds of the components in the materials are investigated by Fourier transform infrared (FT-IR) spectroscopy (Figure S3). In Figure S3a, the composite material of InOF-1@ZIF-67 has the stretching vibration band in the range of 4000-500 cm^{-1}. In particular, the common bands appearing in the materials of InOF-1 and InOF-1@ZIF-67: the distinct bands below 800 cm^{-1} (798 cm^{-1}, 705 cm^{-1}, 652 cm^{-1}, and 592 cm^{-1}) are attributed to the stretching vibration behavior of In-O bonds; The bands of 1559 $cm^{-1}$and 1375 cm^{-1} correspond to the asymmetric and symmetric vibrations of the COO-, respectively; And the peaks of bands at 1677 cm^{-1} and 1618 cm^{-1} are the characteristic absorption of C=N in heterocyclic benzenoid and C=C in benzene ring. Obviously, the spectral stretching vibration band of ZIF-67 appears, in which the typical band at 1000 cm^{-1}, 1145 cm^{-1}, and 1308 cm^{-1} is attributed to the plane vibration of the imidazole ring, and the typical band observed at 1426 cm^{-1} is attributed to the stretching vibration of the imidazole ring. The band vibration observed below 800 cm^{-1} (about 752cm^{-1}) are attributed to the out-of-plane vibration of the imidazole ring. This indicates that the ZIF-67 successfully grows on the surface of InOF-1.

In addition, the chemical conversion of the products after the calcination of InOF-1@ZIF-67 are observed with a noticeable change (Figure S3b). For the samples of CoP-InNC@CNT, CoP-InNC, CoInNC@CNT, and CoInNC, the characteristic adsorption vibration of C=N/C=C after fracture in the imidazole ring or the benzene ring at 1617 cm^{-1}. The band peaks originate from asymmetric vibration and symmetric vibration in -COO at 1551 cm^{-1} and 1380 cm^{-1}, respectively. The peak of the band is attributable to the bending vibration of C-H at 1458 cm^{-1}. Obviously, there is a clear presence in the samples of CoP-InNC@CNT and CoP-InNC that the distinct band peaks at 1081 cm^{-1} and 1042 cm^{-1} are attributed to the vibrational characteristics of Co and P. These results are the alignment with the PXRD characterization results.

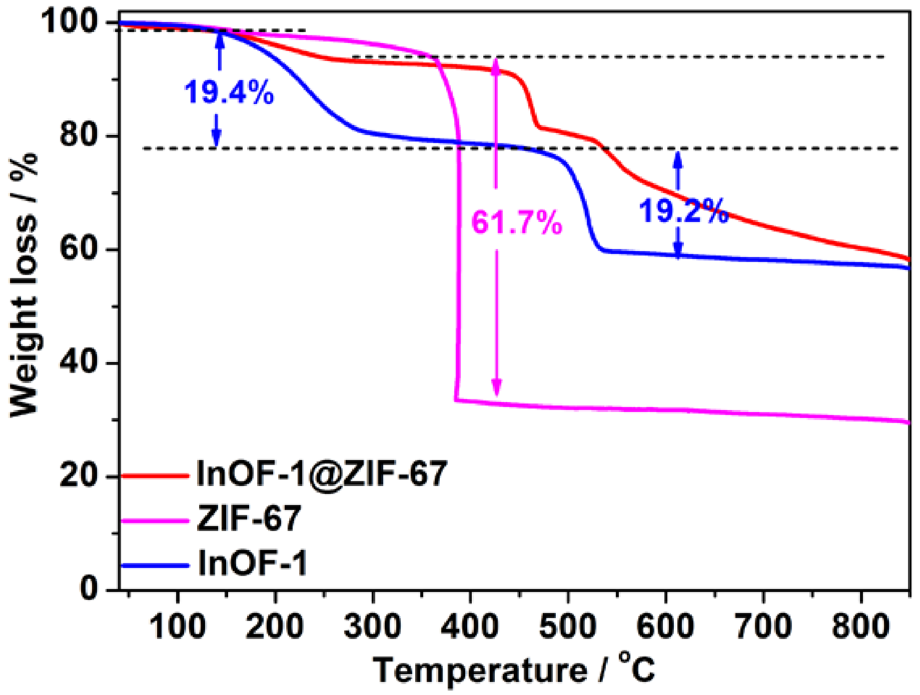

Figure S4. Thermogravimetric analysis (TGA) for InOF-1, ZIF-67, and InOF-1@ZIF-67 in N_2 atmosphere.

For ZIF-67, a sharp weight loss is observed at 380°C that corresponds to the decomposition of the Hmim linker. For InOF-1, the first weight loss in the thermogram (see Figure S4.) corresponds to the sequential loss of the BPTC ligands. The final weight loss corresponds to a complete decomposition of BPTC into In_2O_3 and In. Compared to the initial ZIF-67 and InOF-1, the thermal stability of the InOF-1@ZIF-67 composite has been enhanced.

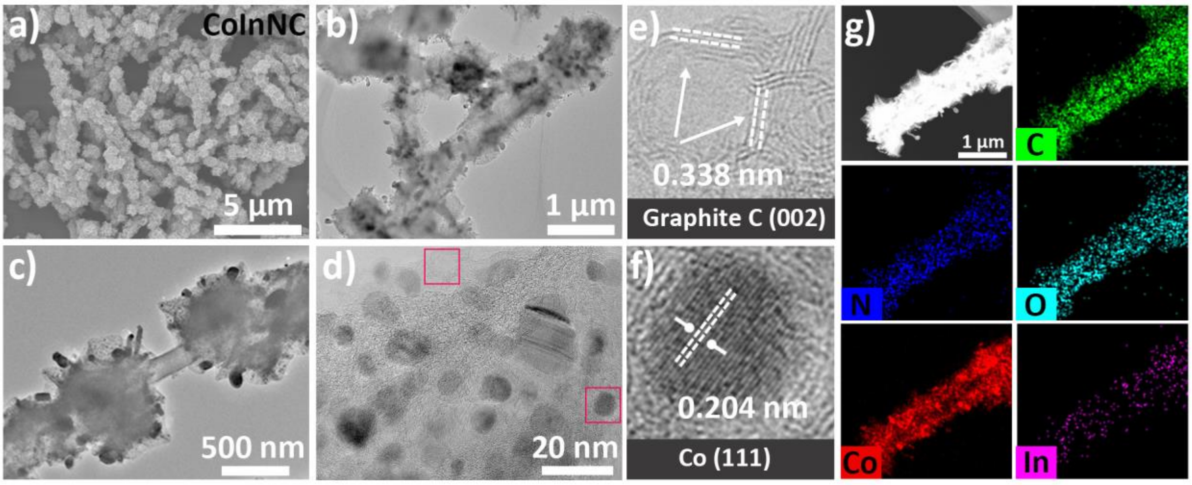

Figure S5. (a) SEM, (b) TEM; (c-d) HR-TEM images at different magnification of the CoInNC micro-rods; (e-f) HR-TEM images of the (d) area, respectively; (g) HAADF-STEM image and the corresponding C, N, O, Co and In mappings of a single CoInNC rod.

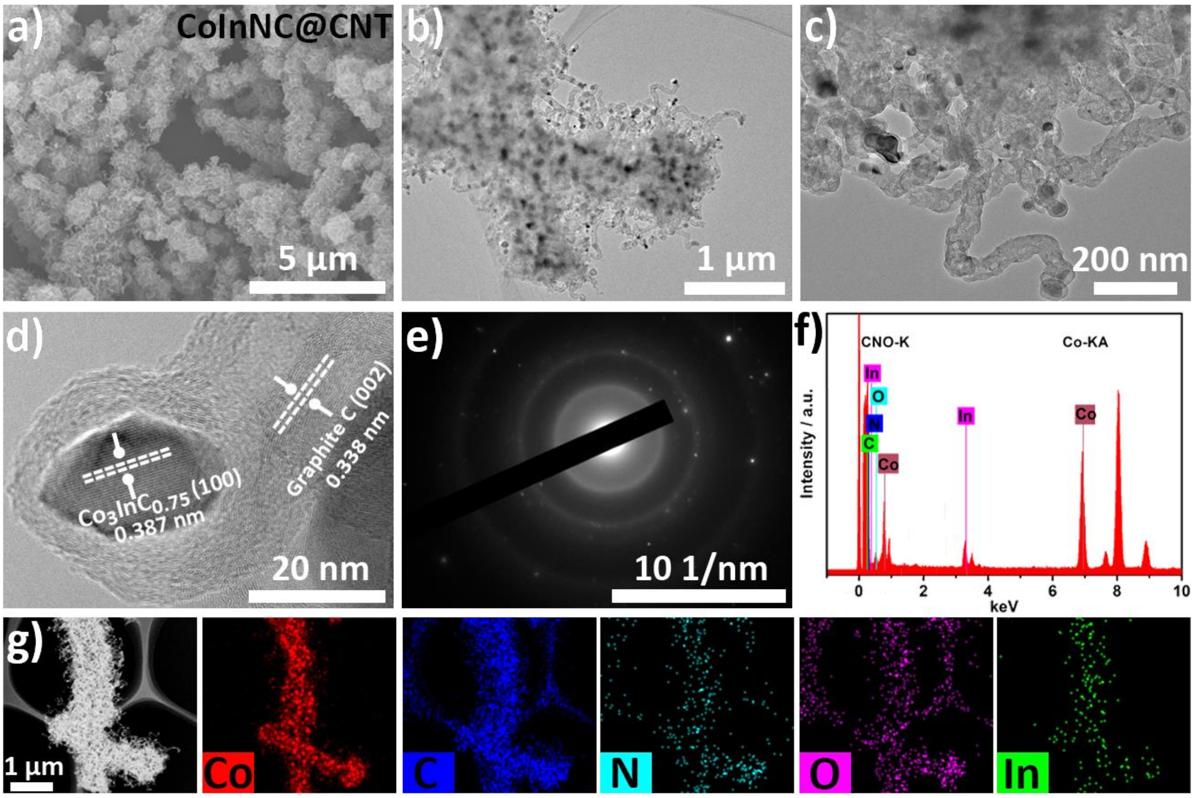

Figure S6. (a) SEM, (b-c) TEM; (d) HR-TEM images at different magnification of the CoInNC@CNT rods; (e) the corresponding SAED pattern; (f) EDX spectrum; (g) HAADF-STEM image and the corresponding C, N, O, Co and In mappings of a single CoInNC rod.

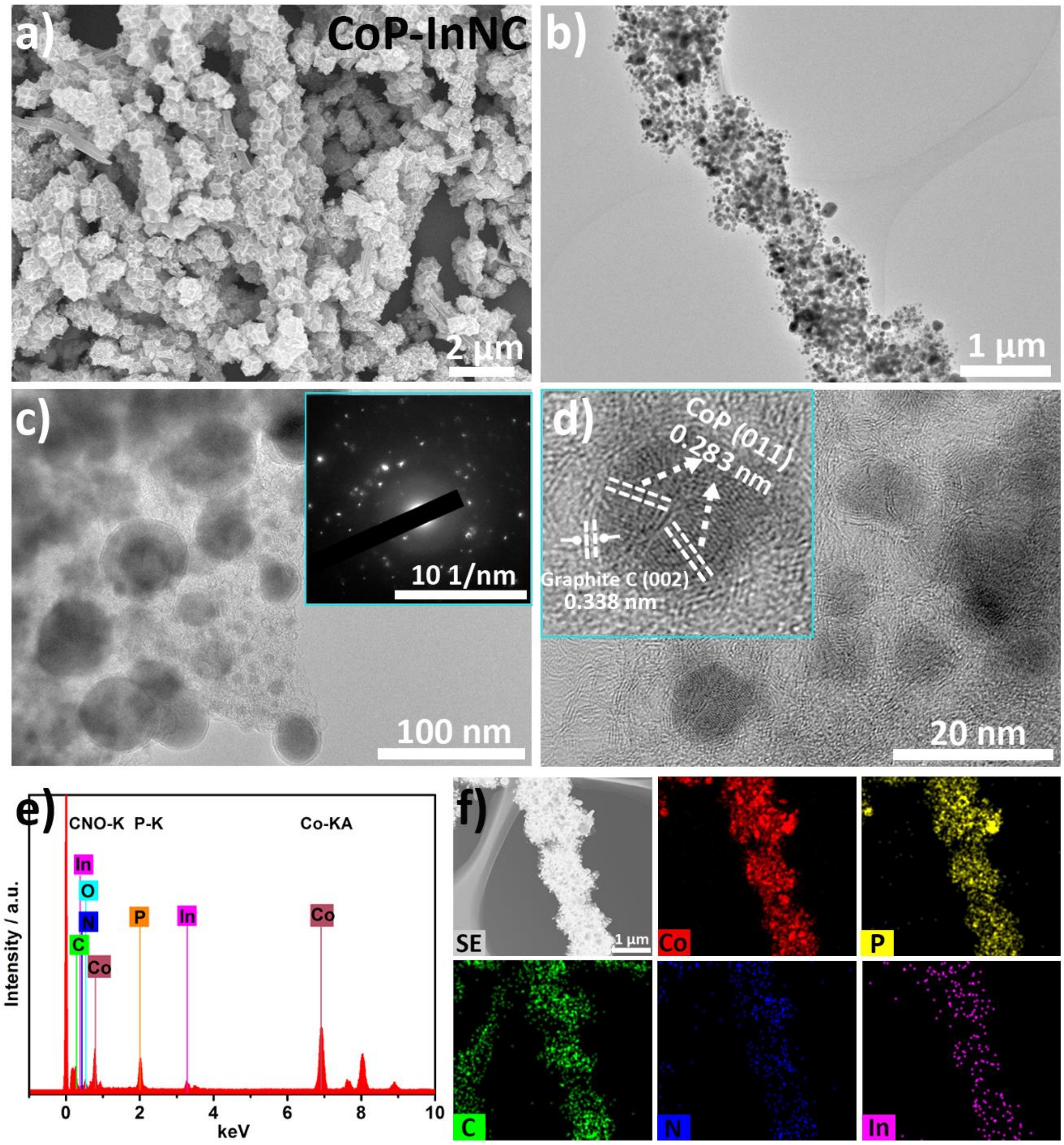

Figure S7. (a) SEM, (b) TEM; (c-d) HR-TEM images at different magnification of the CoP-InNC rods, inset in (c) shows the corresponding SAED pattern; (e) EDX spectrum; (g) HAADF-STEM image and the corresponding C, N, P, Co and In mappings of a single CoP-InNC micro-rod.

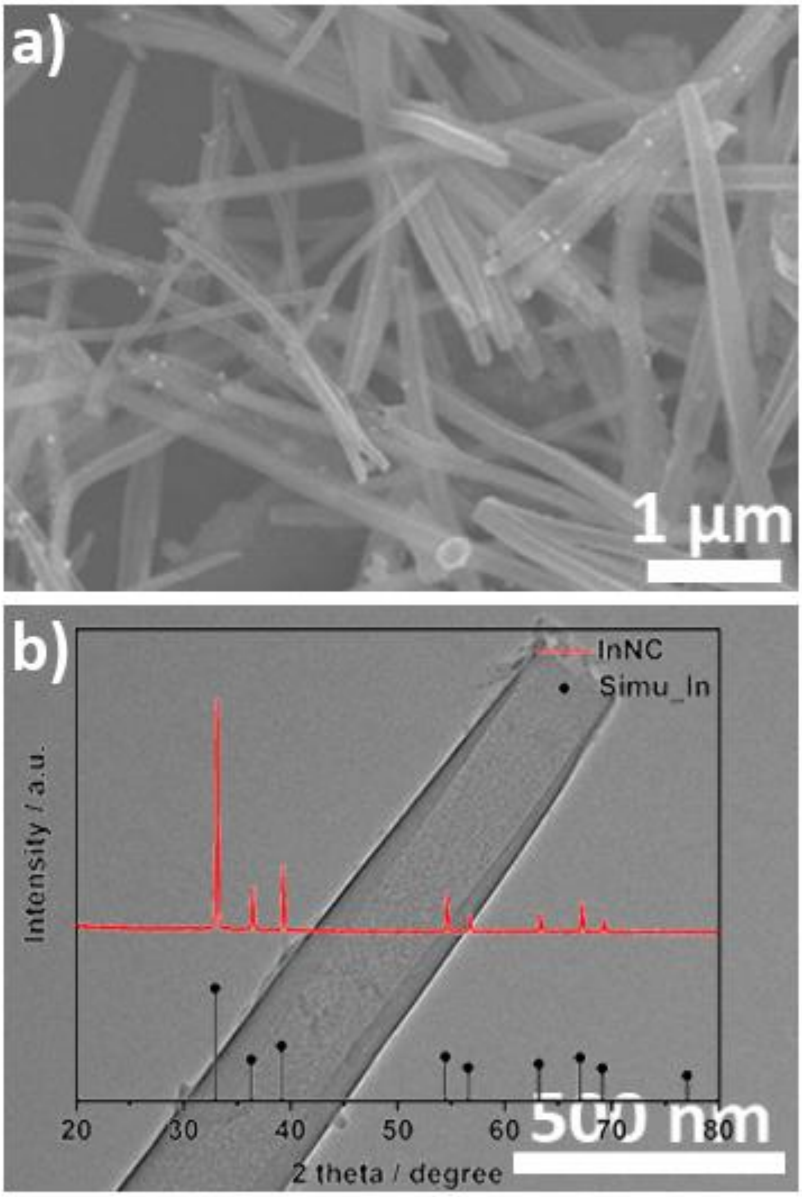

Figure S8. (a) SEM image; (b) TEM image and PXRD pattern of InNC rods.

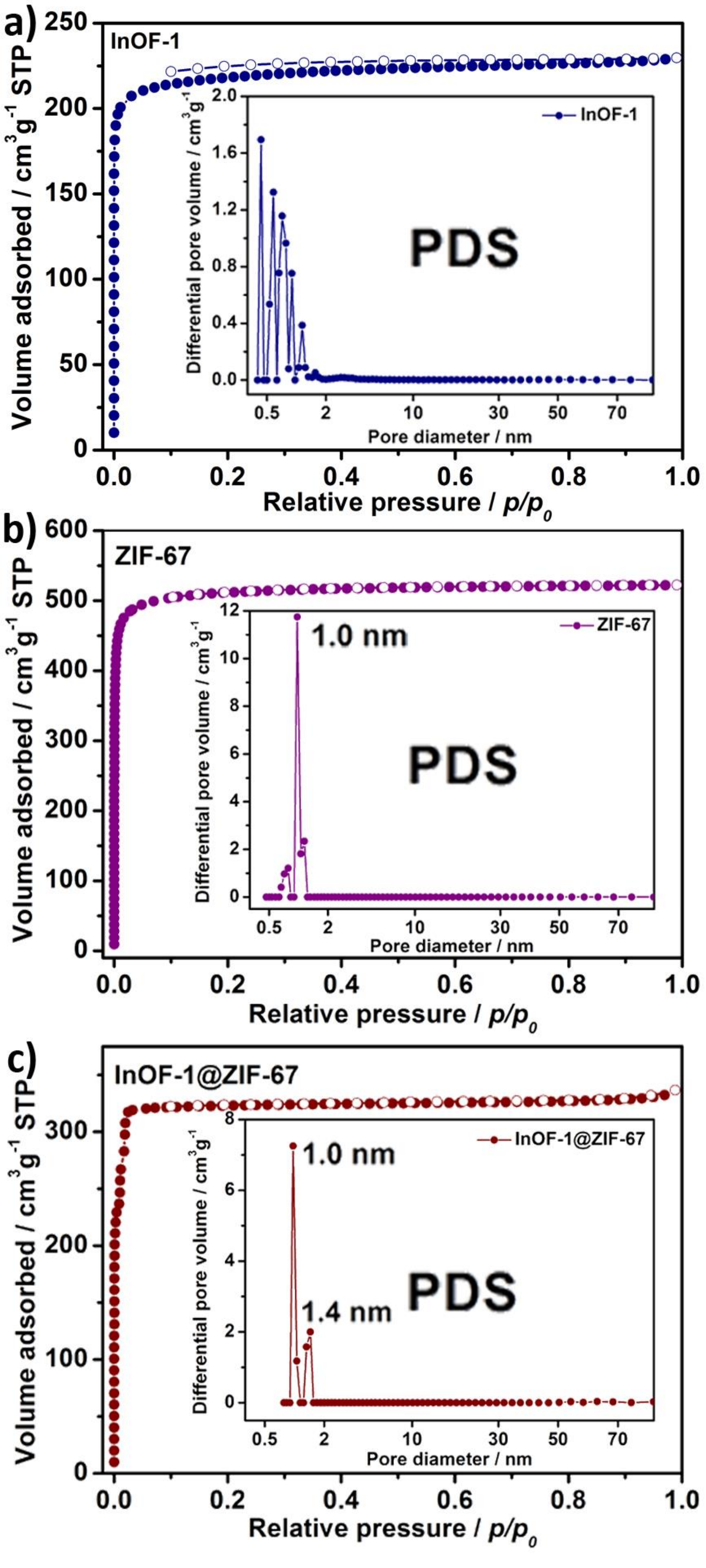

Figure S9. Nitrogen sorption isotherms at 77 K (closed, adsorption; open, desorption) and the corresponding pore size distribution curves of (a) InOF-1, (b) ZIF-67, (c) InOF-1@ZIF-67.

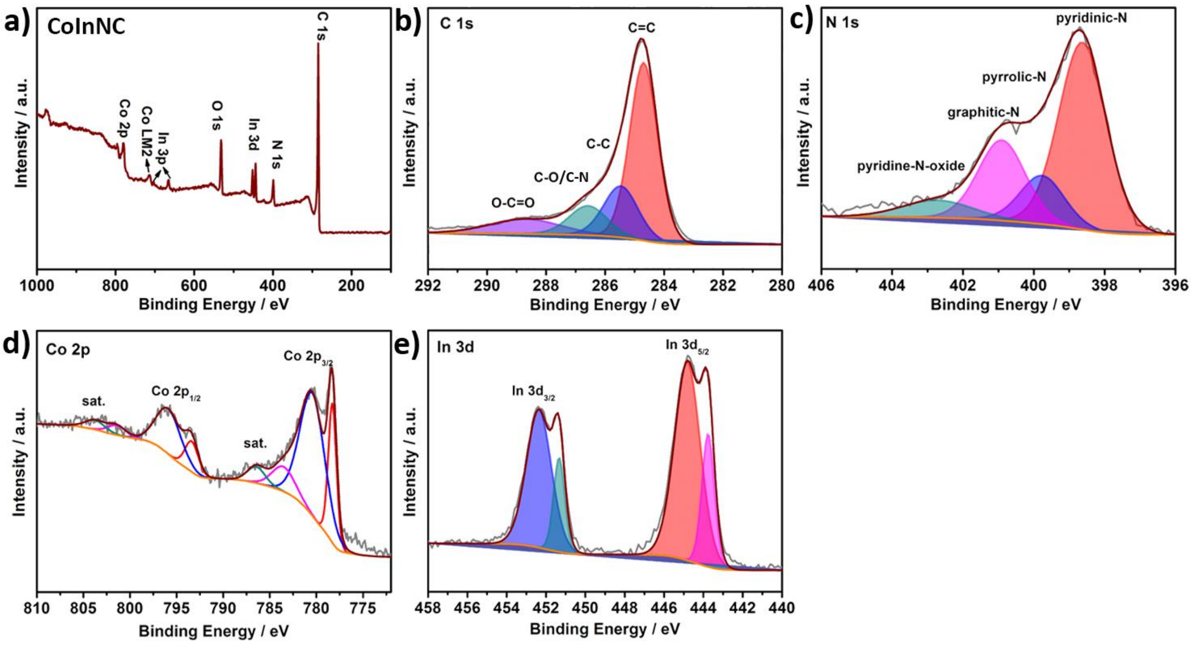

Figure S10. (a) Full survey XPS spectrum of CoInNC rods. b-e) The related C 1s, N 1s, Co 2p, and In 3d fine XPS spectra for CoInNC rods.

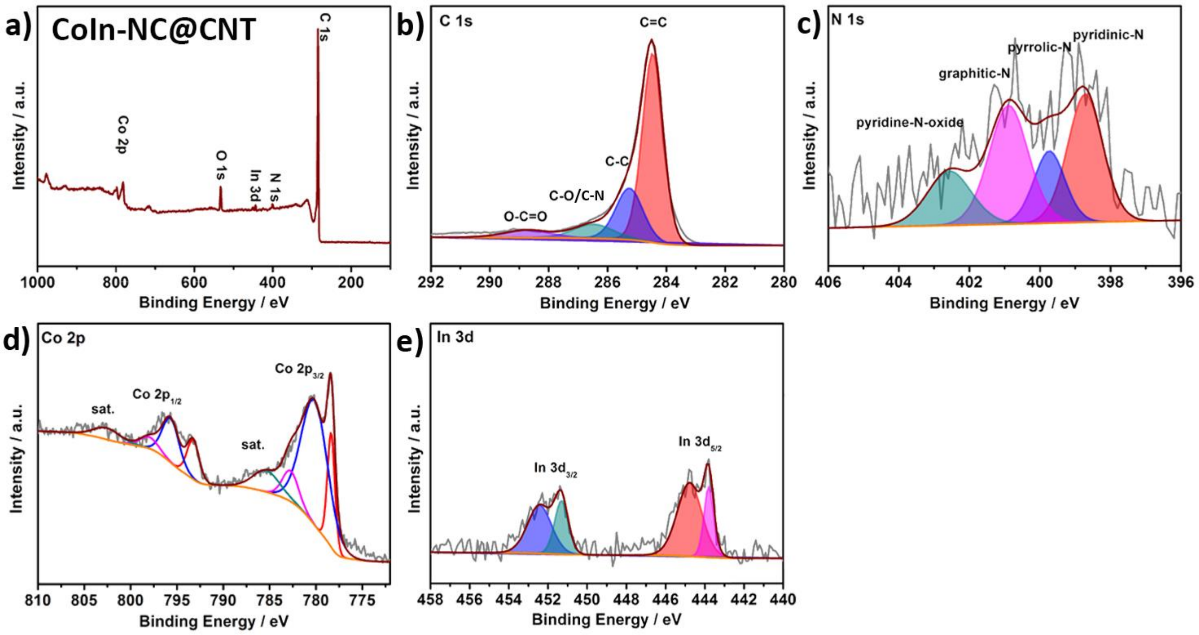

Figure S11. (a) Full survey XPS spectrum of CoInNC@CNT rods. b-e) The related C 1s, N 1s, Co 2p, and In 3d fine XPS spectra for CoInNC rods.

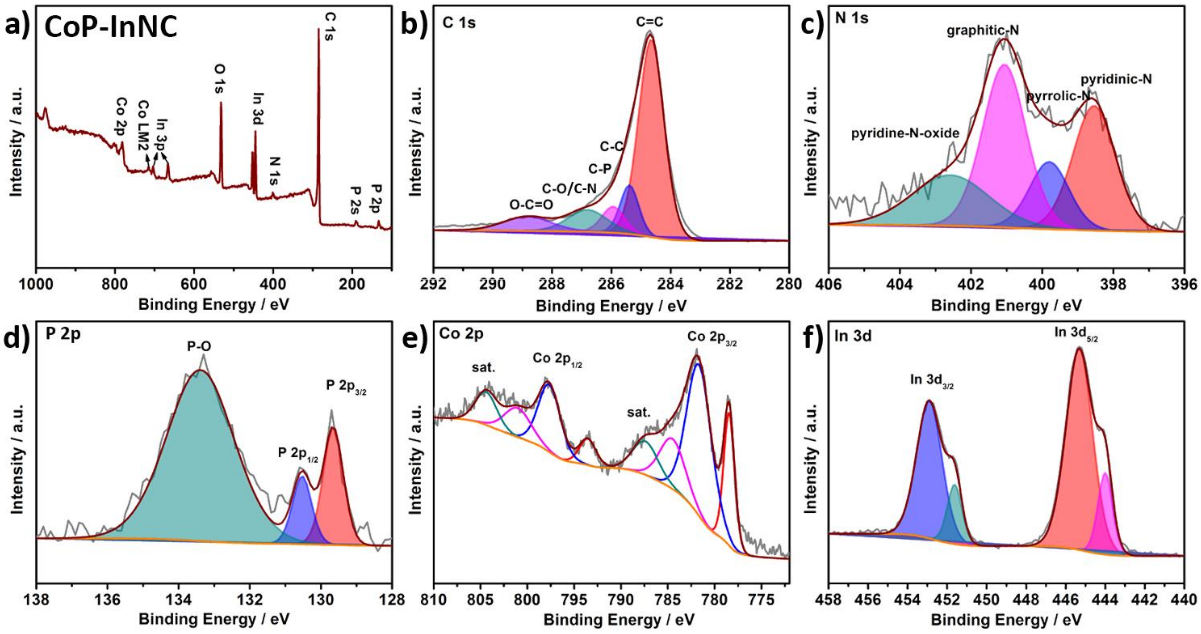

Figure S12. (a) Full survey XPS spectrum of CoP-InNC rods. b-f) The related C 1s, N 1s, P 2p, Co 2p, and In 3d fine XPS spectra for CoP-InNC rods.

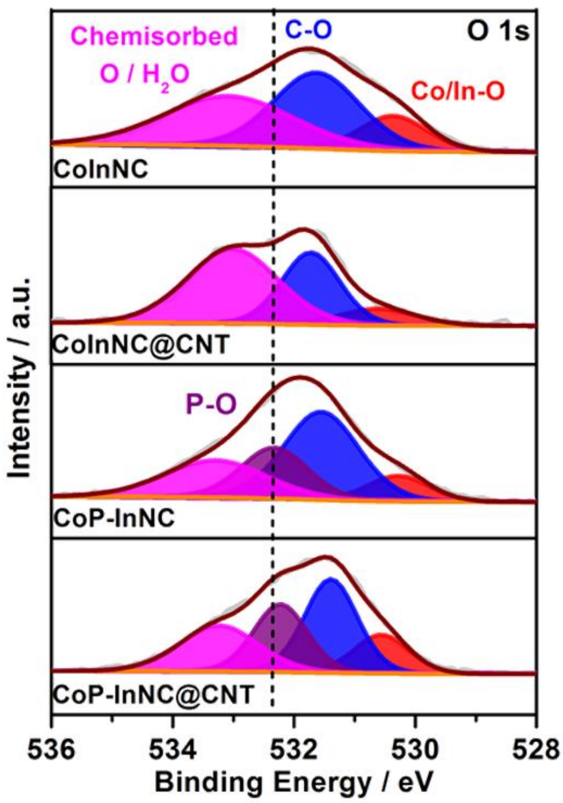

Figure S13. Deconvoluted O 1s spectrum of CoInNC, CoInNC@CNT, CoP-InNC, and CoP-InNC@CNT.

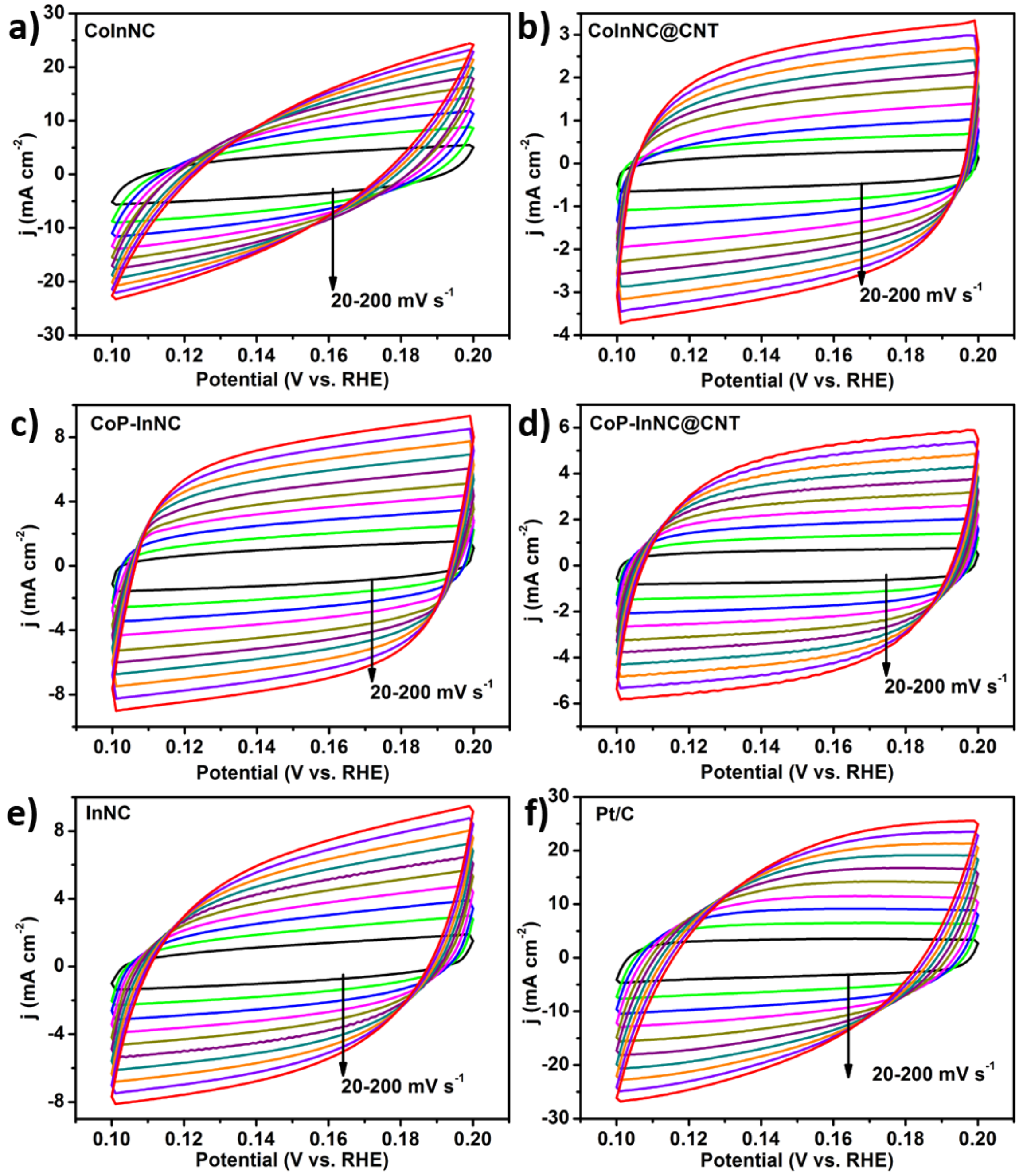

Figure S14. CV plots of the (a) CoInNC, (b) CoInNC@CNT, (c) CoP-InNC, (d) CoP-InNC@CNT, (e) InNC, and (f) Pt/C in 0.5 M H_2SO_4 solution in the region of 0.1~0.2 V vs. RHE for HER.

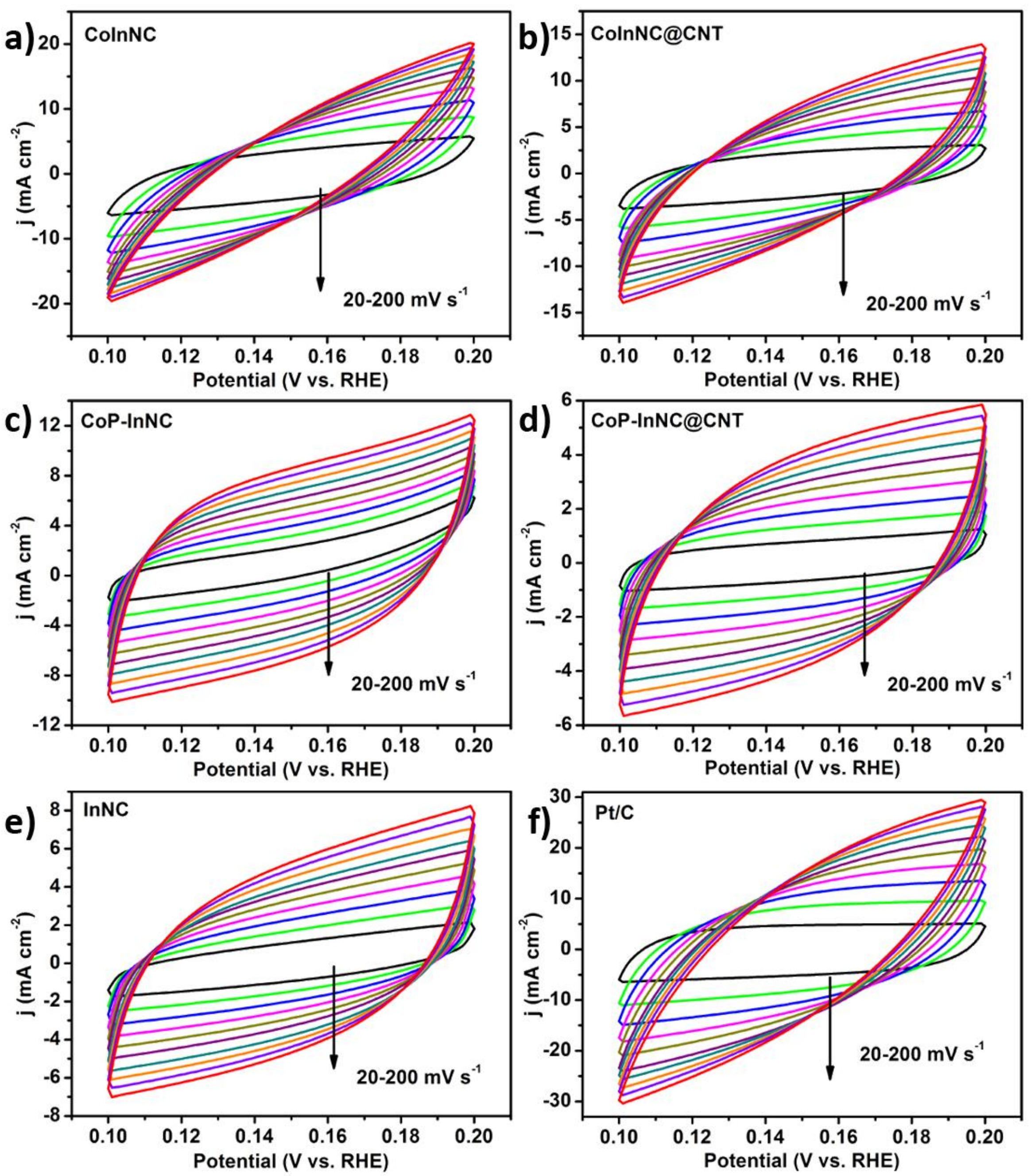

Figure S15. CV plots of the (a) CoInNC, (b) CoInNC@CNT, (c) CoP-InNC, (d) CoP-InNC@CNT, (e) InNC, and (f) Pt/C in 1.0 M KOH solution in the region of 0.1~0.2 V vs. RHE for HER.

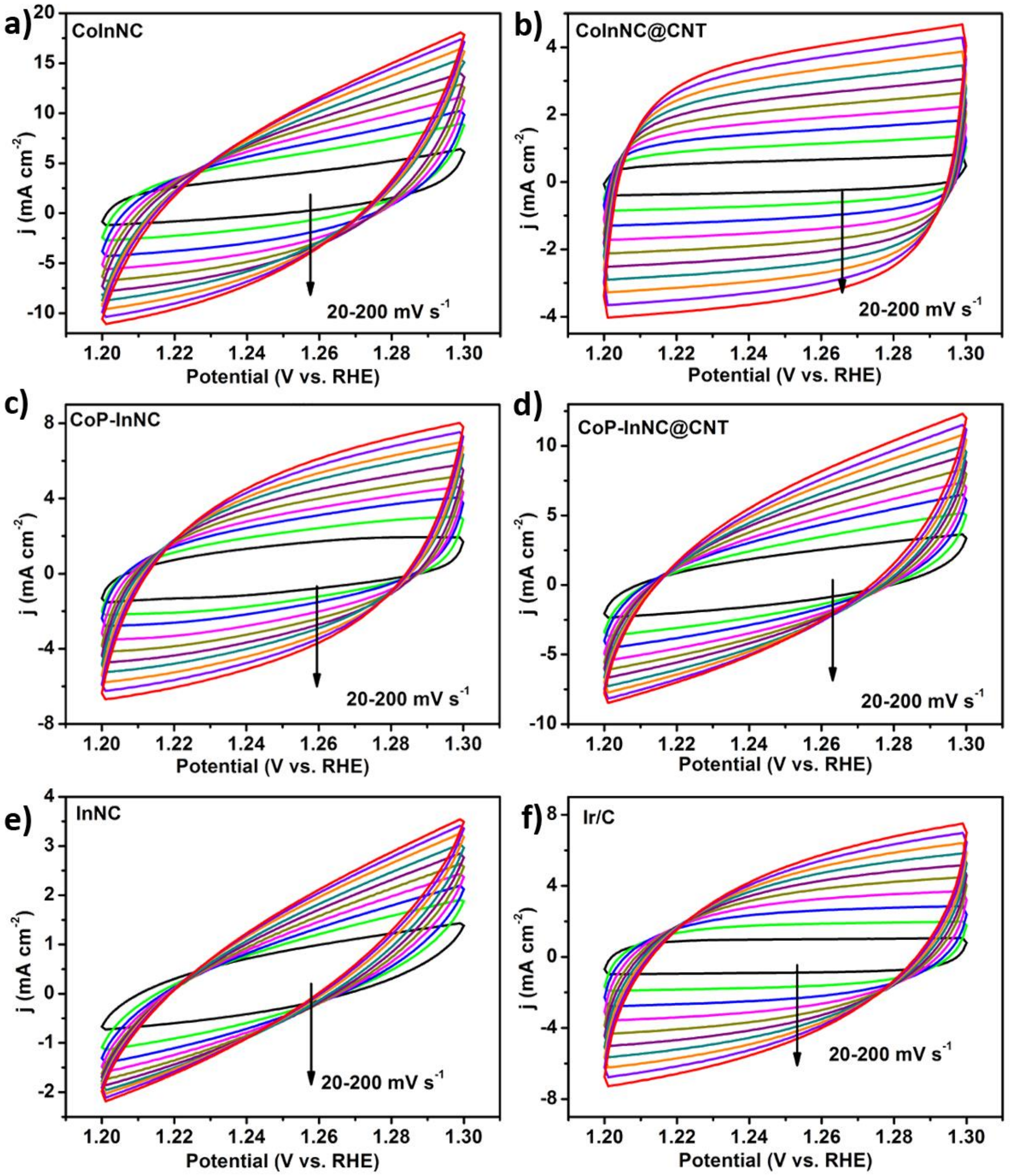

Figure S16. CV plots of the (a) CoInNC, (b) CoInNC@CNT, (c) CoP-InNC, (d) CoP-InNC@CNT, (e) InNC, and (f) Ir/C in 1.0 M KOH solution in the region of 1.20~1.30 V vs. RHE for OER.

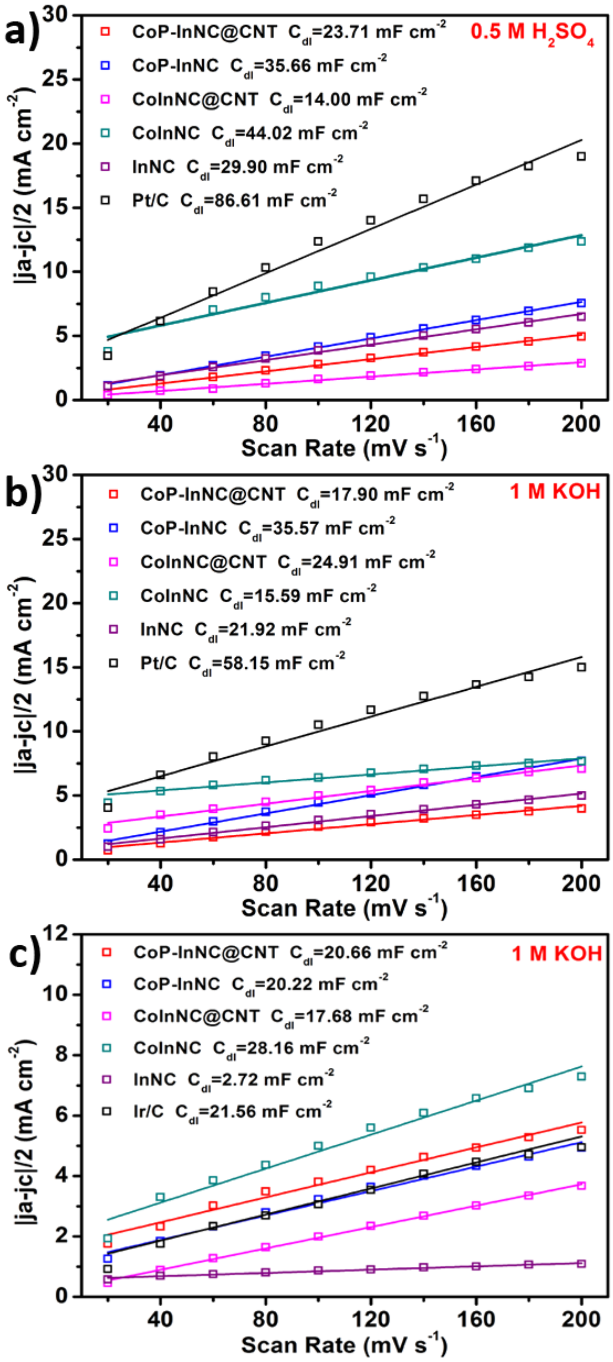

Figure S17. The differences in current density variation plotted against the scan rate fitted to a linear regression enables the estimation of electrochemical double-layer capacitance (C_{dl}) at (a-b) a constant potential of 0.15 V for HER, (c) a constant potential of 1.25 V for OER.

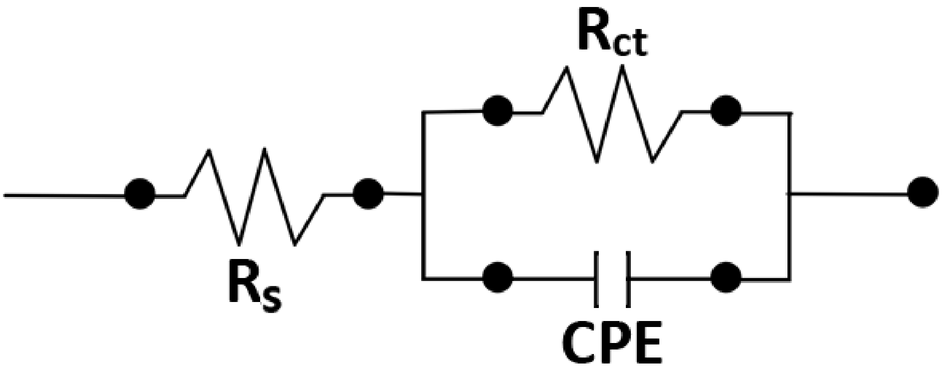

Figure S18 Equivalent electrical circuit used in this work. R_s is the solution resistance, CPE is the element of the catalyst /electrolyte interface, and R_{ct} is the charge transfer resistance at catalyst/electrolyte interface.

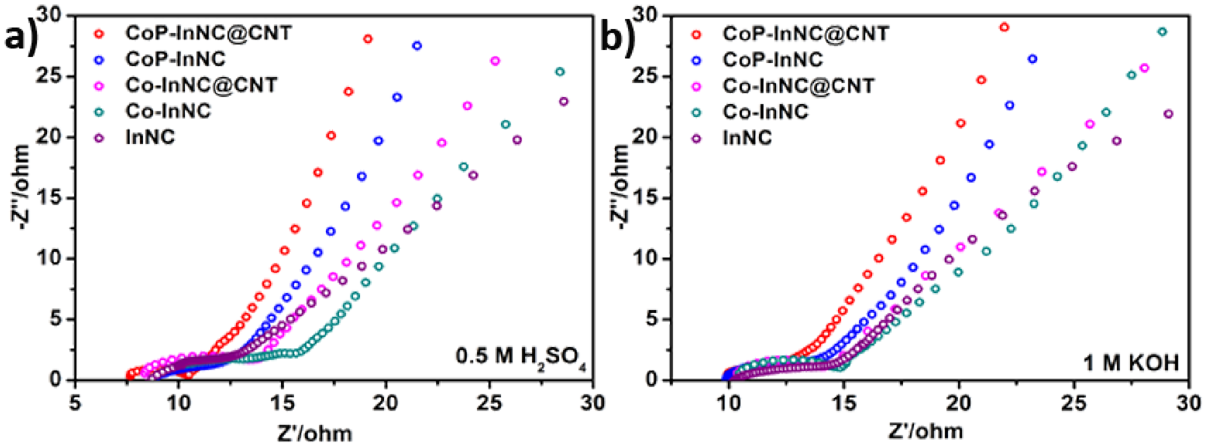

Figure S19. Electrochemical impedance spectroscopy (EIS) Nyquist plots of the CoInNC, CoInNC@CNT, CoP-InNC, CoP-InNC@CNT, and InNC rods in 0.5 M H_2SO_4 solution (a) and 1.0 M KOH solution (b).

**Catalysts**		**CoInNC**	**CoInNC@CNT**	**CoP-InNC**	**CoP-InNC@CNT**	**InNC**
**R_{ct} / Ω**	0.5 M H_2SO_4	7.06	5.47	3.47	3.06	4.21
	1.0 M KOH	4.56	4.07	4.01	2.73	4.30

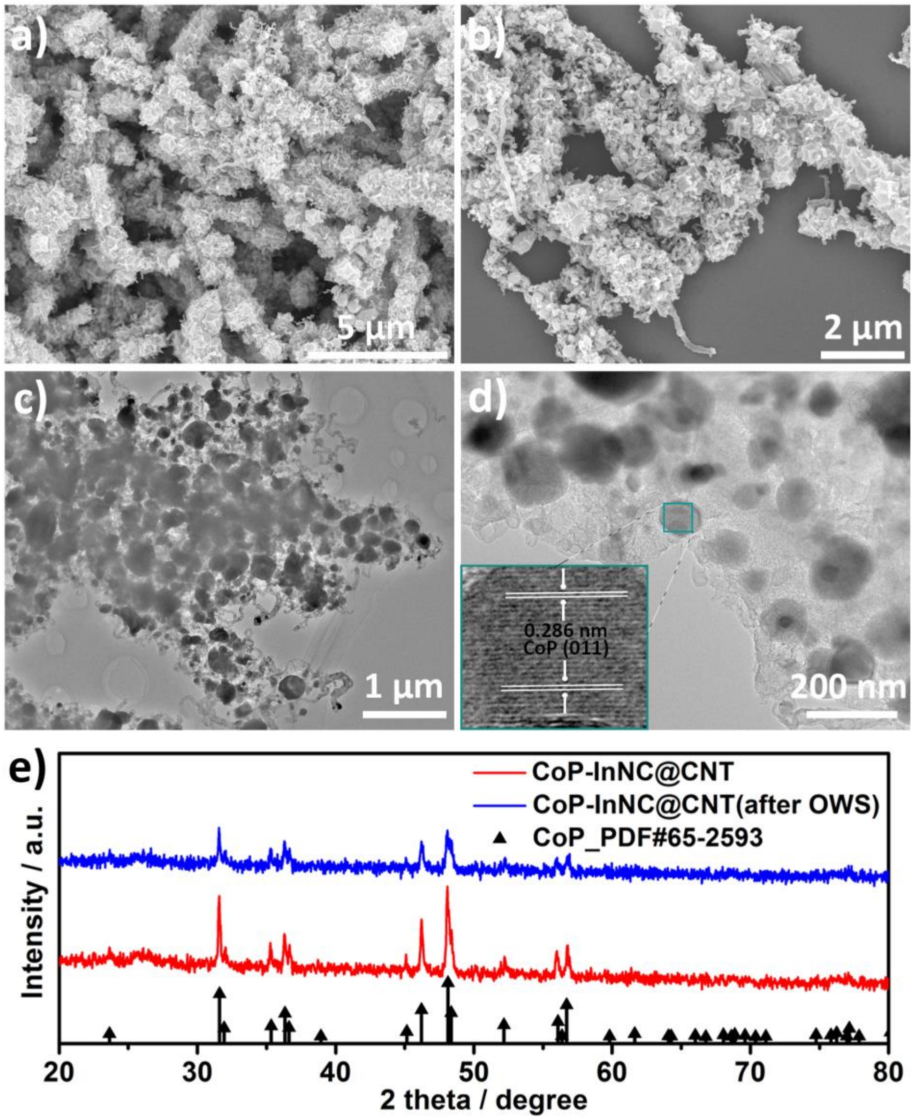

Figure S20. (a, b) SEM and (c, d) TEM images of CoP-InNC@CNT after OWS stability test; (e) PXRD patterns of CoP-InNC@CNT before and after OWS stability test for 15 h.

Table S1. The element content of the as-prepared CoP-InNC@CNT and other control catalysts quantified by EDX.

	**C (wt.%)**	**N (wt.%)**	**O (wt.%)**	**P (wt.%)**	**Co (wt.%)**	**In (wt.%)**
**CoInNC**	72.69	11.89	12.74	-	**2.60**	**0.08**
**CoInNC@CNT**	79.71	10.33	8.02	-	**1.92**	**0.02**
**CoP-InNC**	71.39	11.87	12.60	1.83	**2.26**	**0.05**
**CoP-InNC@CNT**	74.59	9.76	12.15	1.62	**1.87**	**0.01**

Tables S2. Pore characteristics of all samples mentioned in the main article.

**Sample**	**Surface area/m^{2}g^{-1}**		**Total pore volume ^{a}/cm^{3}g^{-1}**	**Micropore volume ^{b}/cm^{3}g^{-1}**
	**BET method**	**Langmuir method**		
**InOF-1**	871	946	0.355381	0.322975
**ZIF-67**	2060	2267	0.807838	0.774763
**InOF-1@ZIF-67**	1282	1419	0.520844	0.493895
**CoInNC**	335	363	0.239966	0.082734
**CoInNC@CNT**	19	21	0.062798	0.006122
**CoP-InNC**	211	227	0.216481	0.039968
**CoP-InNC@CNT**	79	86	0.223539	0.016053

Table S3. Summary of various non-noble metal bifunctional catalysts for HER and OER.

Materials	OER (1.0 M KOH)		HER (0.5 M H_2SO_4)		HER (1.0 M KOH)		Ref.
	η_{10} (mV)	tafel (mV dec^{-1})	η_{10} (mV)	tafel (mV dec^{-1})	η_{10} (mV)	tafel (mV dec^{-1})	
CoP-InNC@CNT	270	84	153	62	159	56	This work
CoP-InN	330	93	195	65	177	78	
CoP/NCNHP	310	70	140	53	115	66	S1
CoP/PNC	300	77	99	46	165	70	S2
CoP@PC-750	283	53	72	49	76	52	S3
Hollow Mo-CoP nanoarrays	305	56	-	-	40	65	S4
CoP/Co_2P	317	58.9	99	51.4	103	61.2	S5
$Co_{0.6}Fe_{0.4}P$-1.125	298	48	97	-	133	61	S6
Ni_2P nanosheets	320	105	-	-	168	63	S7
CoP/rGO-400	340	66	105	50	150	38	S8
CoP/MoP@NC	270	81	-	-	94	40	S9

Table S4. Comparison of electrocatalytic performance for various transition metal-based electrodes for the overall water splitting at 10 mA cm^{-2}.

Electrolyzer		η_{10} (V)	substrate	Ref.
**Anode**	**Cathode**			
**CoP-InNC@CNT**	**CoP-InNC@CNT**	1.58	carbon cloth	**This work**
**Ir/C**	**Ir/C**	1.52	carbon cloth	**This work**
**Co/CoP-5**	**Co/CoP-5**	1.45	Ni foam	S10
**Ni-Co-P HNBs**	**Ni-Co-P HNBs**	1.62	Ni foam	S11
**FeP/Ni_2P**	**FeP/Ni_2P**	1.42	carbon paper	S12
**CoP/NCNHP**	**CoP/NCNHP**	1.64	carbon paper	S1
**CoP@NiFe–OH/SPNF**	**CoP@NiFe–OH/SPNF**	1.53	Ni foam	S13
**FeNiP/NCH**	**FeNiP/NCH**	1.59	Ni foam	S14
**Fe-CoP UNSs**	**Fe-CoP UNSs**	1.46	Ni foam	S15
**CoP-Co_2P@PC/PG**	**CoP-Co_2P@PC/PG**	1.567	Ni foam	S16
**CoP-MNA**	**CoP-MNA**	1.62	Ni foam	S17
**NiCoP/NF**	**NiCoP/NF**	1.58	Ni foam	S18
**Co_3O_4-MTA**	**Co_3O_4-MTA**	1.63	Ni foam	S19

References

[S1] Y. Pan, K. A. Sun, S. J. Liu, X. Cao, K. L. Wu, W.-C. Cheong, Z. Chen, Y. Wang, Y. Li, Y. Q. Liu, D. S. Wang, Q. Peng, C. Chen, Y. D. Li, *J. Am. Chem. Soc.* **2018**, *140*, 2610.

[S2] Z. Peng, Y. Yu, D. Jiang, Y. L. Wu, B. Y. Xia, Z. H. Dong, *Carbon* **2019**, *144*, 464.

[S3] J. D. Wu, D. P. Wang, S. A. Wan, H. L. Liu, C. Wang, X. Wang, *Small* **2019**, 1900550.

[S4] C. Guan, W. Xiao, H. J. Wu, X. M. Liu, W. J. Zang, H. Zhang, J. Ding, Y. P. Feng, S. J. Pennycook, J. Wang, *Nano Energy 2018*, **48**, 73.

[S5] L. Y. Chen, Y. Y. Zhang, H. F. Wang, Y. X. Wang, D. Z. Li, C. Y. Duan, *Nanoscale* **2018**, *10*, 21019.

[S6] Y. B. Lian, H. Sun, X. B. Wang, P. W. Qi, Q. Q. Mu, Y. J. Chen, J. Ye, X. H. Zhao, Z. Deng, Y. Peng, *Chem. Sci*. **2019**, *10*, 464.

[S7] Q. Wang, Z. Q. Liu, H. Y. Zhao, H. Huang, H. Jiao, Y. P. Du, *J. Mater. Chem. A* **2018**, *6*, 18720.

[S8] L. Jiao, Y.-X. Zhou, H.-L. Jiang, *Chem. Sci.* **2016**, *7*, 1690.

[S9] Y.-J. Tang, H.-J. Zhu, L.-Z. Dong, A.-M. Zhang, S.-L. Li, J. Liu, Y.-Q. Lan, *Appl. Catal. B: Environ.* **2019**, *245*, 528.

[S10] Z.-H. Xue, H. Su, Q.-Y. Yu, B. Zhang, H.-H. Wang, X.-H. Li, J.-S.Chen, *Adv. Energy Mater.* **2017**, *7*, 1602355.

[S11] E. L. Hu, Y. F. Feng, J. W. Nai, D. Zhao, Y. Hu, X. W. (David) Lou, *Energy Environ. Sci.* **2018**, *11*, 872.

[S12] F.Yu, H. Q. Zhou, Y. F. Huang, J. Y. Sun, F. Qin, J. M. Bao, W. A. Goddard III, S. Chen, Z. F. Ren, *Nat. Commun.* **2018**, *9*, 2551.

[S13] Y. Li, S. W. Guo, T. Jin, Y. L. Wang, F. Y. Cheng, L. F. Jiao, *Nano Energy* **2019**, *63*, 103821.

[S14] Y.-S. Wei, M. Zhang, M. Kitta, Z. Liu, S. Horike, Q. Xu, *J. Am. Chem. Soc*. **2019**, *141*, 7906.

[S15] Y. Li, F. M. Li Y. Zhao, S.-N. Li, J.-H. Zeng, H.-C. Yao, Y. Chen, *J. Mater. Chem. A* **2019**, *7*, 20658.

[S16] J. Yang, D. H. Guo, S. L. Zhao, Y. Lin, R. Yang, D. D. Xu, N. E. Shi, X. S. Zhang, L. Z. Lu, Y.-Q. Lan, J. C. Bao, M. Han, *Small* **2019**, *15*, 1804546.

[S17] Y.-P. Zhu, Y.-P. Liu, T. Z. Ren, Z.-Y. Yuan, *Adv. Funct. Mater.* **2015**, *25*, 7337.

[S18] H. F. Liang, A. N. Gandi, D. H. Anjum, X. B. Wang, U. Schwingenschlögl, H. N. Alshareef, *Nano Lett.* **2016**, *16* (12), 7718.

[S19] Y. P. Zhu, T. Y. Ma, M. Jaroniec, S. Z. Qiao, *Angew. Chem. Int. Ed.* **2017**, *56*, 1324.
